# Molecular mechanism of fungal effector PevD1-NRP interaction and discovery of a small-molecule disruptor

**DOI:** 10.1016/j.jbc.2026.113431

**Published:** 2026-08-10

**Authors:** Xin Yang, Siyuan Tian, Chao Xu, Lu Liu, Fang Bai, Xianglei Zhang

**Affiliations:** 1 Shanghai Institute for Advanced Immunochemical Studies and School of Life Science and Technology, ShanghaiTech University, Shanghai, China; 2 Lingang Laboratory, Shanghai, China; 3 Shanghai Clinical Research and Trial Center, Shanghai, China

**Keywords:** molecular mechanism, NRP, PevD1, verticillium dahliae

## Abstract

Verticillium wilt, caused by *Verticillium dahliae*, is a devastating vascular disease that severely affects cotton and other dicotyledonous crops worldwide. The fungal effector protein PevD1 promotes disease progression by interacting with the host asparagine-rich protein (NRP) and disrupting cryptochrome 2 (CRY2)-mediated signaling; however, the molecular basis of this interaction remains poorly understood. Here, we combined computational modeling, molecular interaction analysis, mutagenesis, and structural biology approaches to investigate the PevD1-NRP-CRY2 regulatory mechanism in *Arabidopsis thaliana*. AlphaFold-Multimer prediction and molecular dynamics (MD) simulations indicated that both PevD1 and the photolyase homology region of CRY2 interact with the death-associated domain of NRP through highly overlapping interfaces. Surface plasmon resonance and isothermal titration calorimetry analyses showed that PevD1 binds NRP with substantially higher affinity than CRY2 (*K*_D_ values of 0.19 μM and 4.34 μM, respectively). Site-directed mutagenesis further identified E108 and L111 of PevD1 as critical residues required for NRP recognition. We subsequently determined the crystal structure of PevD1 at 1.35 Å resolutions and performed structure-guided virtual screening targeting the predicted interaction interface. This led to the identification of compound A6, which binds PevD1, forms a key interaction with residue E108, and disrupts the PevD1-NRP interaction in biochemical assays. Together, these findings define the molecular mechanism underlying the PevD1-NRP-CRY2 interaction and establish a framework for the development of effector-targeting anti-virulence compounds against *V. dahliae*.

Fungal infectious diseases represent a critical challenge to global food security, causing substantial yield reductions in staple crops such as rice, maize, and wheat (1). These pathogens inflict severe economic losses on agricultural systems worldwide (2, 3). Over the past 2 decades, the expansion of international trade, increased transportation, and climate change have significantly accelerated the spread of fungal plant pathogens across regions (4, 5, 6). Furthermore, fungi exhibit remarkable environmental adaptability, enabling rapid evolutionary responses to changing conditions and the development of resistance to antifungal agents (7, 8, 9).

*Verticillium dahliae* is a soilborne fungal pathogen that infects plant roots and spreads, causing Verticillium wilt in over 400 dicotyledonous plant species (10). This pathogen significantly impacts economically vital crops, including cotton and tobacco, leading to severe agricultural losses (11). Infected plants exhibit characteristic symptoms such as leaf chlorosis, wilting, growth retardation, and eventual plant death (12). Pathogenicity is mediated through the secretion of effector proteins, including PevD1 (Protein elicitor from *V. dahliae* 1), a protein previously isolated from *V. dahliae* culture filtrates, has been identified as an elicitor-like molecule capable of inducing defense responses in tobacco plants (13). While in cotton, PevD1 specifically inhibits the antifungal activity of GhPR5, a key defense-related protein, thereby compromising the host's resistance mechanisms (14). Additionally, in Arabidopsis and cotton, PevD1 targets the core senescence-regulating transcription factor ORE1, inducing leaf senescence and further promoting fungal infection while accelerating the plant's aging process (15).

Several studies have revealed that PevD1-mediated disease resistance in tobacco is regulated by asparagine-rich protein (NRP) (16, 17, 18). Furthermore, overexpression of PevD1 in Arabidopsis has been shown to accelerate flowering through its interaction with NRP, with *V. dahliae*-infected plants exhibiting earlier flowering compared to uninfected controls (19). These findings suggest that PevD1 plays a role in modulating plant developmental processes during *V. dahliae* infection. Subsequently, the in-depth research discovered that during severe infection by the fungal pathogen *V. dahliae*, the effector protein PevD1 competitively binds to NRP, displacing CRY2 (Arabidopsis cryptochrome 2) and facilitating its nuclear translocation (16). This aberrant signaling cascade induces premature flowering, disrupts developmental homeostasis, and triggers leaf chlorosis and wilting, ultimately leading to plant mortality (20, 21, 22, 23, 24). While the PevD1-NRP-CRY2 pathway has been implicated in Verticillium wilt pathogenesis, the precise molecular interactions governing this tripartite mechanism remain unresolved. Additionally, no commercially available fungicides specifically targeting *V. dahliae* have been developed to date, highlighting an unmet need for effective disease management strategies.

In this study, we employed an integrated multidisciplinary approach, combining computational, molecular, and structural biology techniques to systematically investigate the binding interactions of PevD1 and CRY2 with the development and cell death (DCD) domain of the NRP. Experimental analyses demonstrate significant spatial overlap at the binding interface, with measured *K*_D_ values of 0.19 μM and 4.34 μM, respectively. Through site-directed mutagenesis, we identified E108 and L111 of the PevD1 protein as critical residues mediating its interaction with NRP. Based on the high-resolution crystal structure of PevD1, we performed virtual screening targeting the PevD1-NRP interaction interface and discovered compound A6, which specifically binds to PevD1 and forms stable hydrogen bonds with the key residue E108, effectively inhibiting the interaction of the PevD1-NRP. This study represents the first comprehensive elucidation of the molecular interaction mechanism within the PevD1-NRP-CRY2 signaling pathway, laying an important theoretical foundation for the development of novel fungicides targeting the PevD1 protein of *V. dahliae*.

## Results

### Structural prediction of the PevD1-NRP and NRP-CRY2 complexes

To elucidate the molecular mechanisms underlying the interaction between PevD1, CRY2, and NRP in Arabidopsis, we predicted the models of PevD1-NRP and CRY2-NRP complexes using AlphaFold-Multimer. Predicted Aligned Error (PAE) maps serve as a quantitative measure of inter-residue distance prediction confidence, where lower PAE values correlate with higher interaction stability. Analysis of the CRY2-NRP complex (Fig. S1, *A* and *B*) revealed that stable interactions were predominantly localized to well-ordered domains. Because the PAE values were increased in the disordered regions of CRY2, indicating reduced confidence in their predicted binding interfaces with NRP. CRY2 consists of two domains: an ordered photolyase homology region (PHR) and a disordered CRY C-terminal extension domain (25). In NRP, the structured region corresponds to the DCD domain (26). Our predictions suggest that CRY2 interacts with NRP *via* its PHR domain, whereas PevD1 also targets NRP through the DCD domain. Notably, the interaction between PevD1 and the DCD domain has been experimentally validated, supporting our prediction (16). A parallel analysis of the PevD1-NRP complex demonstrated that molecular interactions are similarly restricted to the structured regions (Fig. S2).

**Figure 1:**
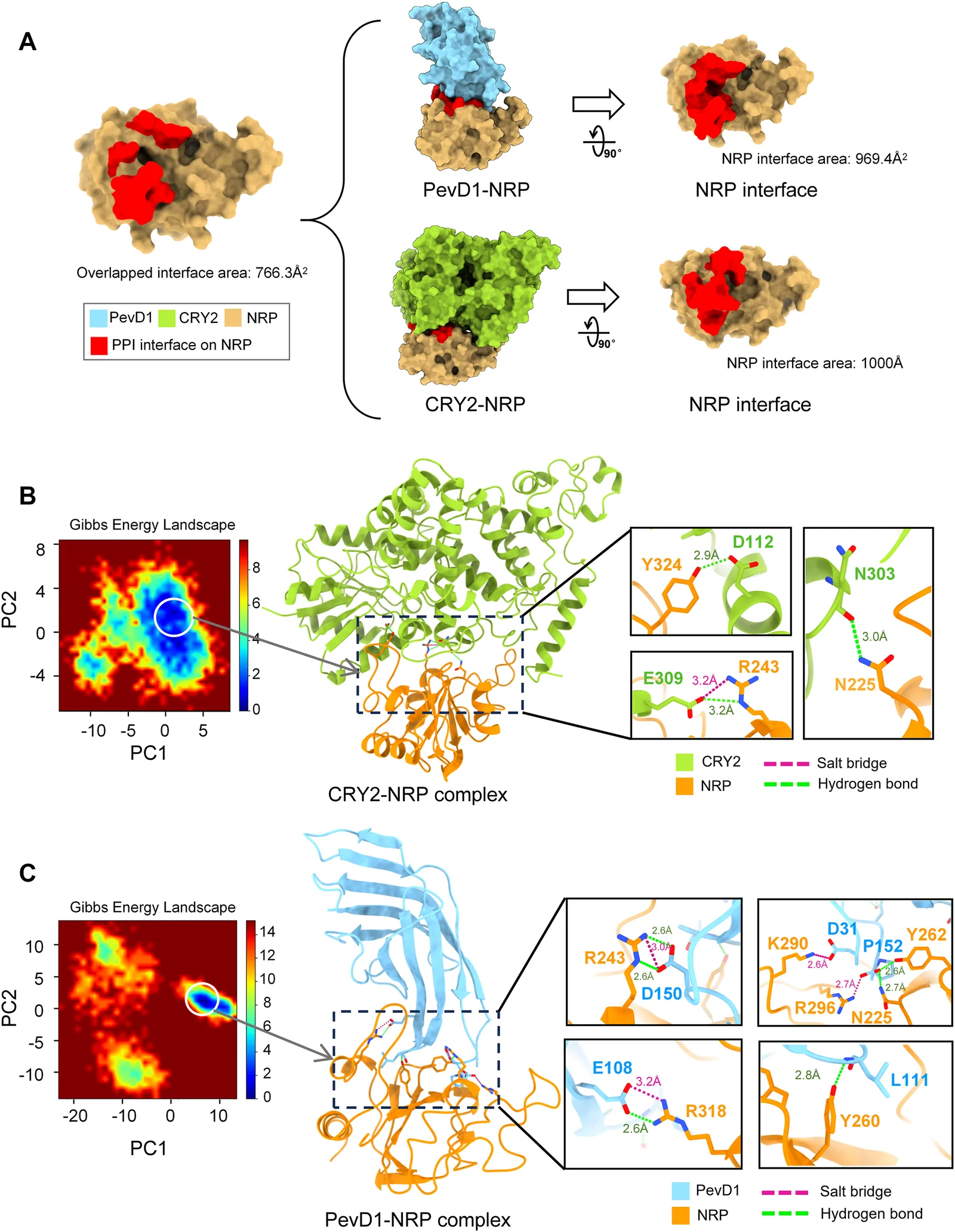
**Predicted protein binding modes of PevD1 and CRY2 with NRP.***A,* the predicted binding modes of CRY2-NRP and PevD1-NRP were determined using AF-Multimer. The *right panel* shows the predicted binding modes for both complexes, and NRP for each complex is displayed as surface, with the interaction interfaces highlighted in *red*. The *left panel* illustrates the overlapping protein interaction interfaces in both NRP complex interfaces, also marked in *red*. *B and C,* Molecular dynamics (MD) simulations were conducted for the CRY2-NRP *(B)* and PevD1-NRP *(C)* complexes, with the lowest-energy conformations along the trajectory identified *via* free energy landscape maps. The structures correspond to the stable conformations obtained after MD simulations, highlighting the residues that remain stably positioned at the interaction interfaces and the distance of interacting residues at the freeze frame of MD. NRP, asparagine-rich protein; CRY2, cryptochrome 2.

Structural analysis of the predicted complex models suggested that CRY2 and PevD1 share an overlapping predicted binding site on NRP (Fig. 1*A*). To further examine the physicochemical properties of these interfaces, electrostatic potential maps were generated using the APBS electrostatics plugin in PyMOL. These analyses revealed complementary charge distributions at the predicted CRY2-NRP and PevD1-NRP interfaces (Fig. S1*C*), providing additional support for the plausibility of the predicted interaction models. Then we assessed the structural stability and dynamic behavior of the two complexes. For each model of CRY2-NRP and PevD1-NRP, three parallel molecular dynamics (MD) simulations spanning 200 nanoseconds were conducted, with 1000 trajectory frames generated per simulation. RMSD of complex for each trajectory is showed in Figure S1*D*. Principal component analysis performed on the MD trajectories further elucidated the conformational changes occurring at the interaction interface. Utilizing the Principal component analysis -derived data, Gibbs free energy landscapes (FELs) were constructed for both complexes throughout the MD simulations. These landscapes were analyzed to identify the energetically optimized conformation of each complex, corresponding to the lowest-energy regions on the free energy surface (Fig. 1, *B* and *C*). In addition, we performed a comprehensive statistical analysis of interaction pairs between protein complexes across all trajectory frames, with a focus on identifying persistently stable residue interactions at the binding interface.

Protein-protein interactions (PPIs) were systematically recorded in every frame of three independent MD trajectories. Interaction pairs observed in at least 70% of the trajectory frames were classified as stable and deemed structurally critical for complex formation. The results indicate that the CRY2-NRP complex exhibits multiple stable interactions at its binding interface. Specifically, residues E309, N303 and D112 of CRY2 form hydrogen bonds with R242, N224 and Y323 of NRP, respectively, while E309 of CRY2 engages in a salt bridge interaction with R242 of NRP (Fig. 1*B*). In contrast, the PevD1-NRP complex demonstrates distinct binding characteristics. Residues P152 and L111 of PevD1 form stable hydrogen bonds with N224, Y261, and Y259 of NRP. Notably, PevD1 exhibits enhanced electrostatic interactions compared to CRY2, with residues D150, P152, and E108 forming additional salt bridges with R242, R295, and R317 of NRP. Besides, D31 of PevD1 and K289 of NRP also form a salt bridge at the energy-minimized conformation (Fig. 1*C*). Together, these analyses indicate that the PevD1-NRP complex contains a greater number of stable interaction pairs than the CRY2-NRP complex, which may contribute to the higher binding affinity of PevD1 for NRP. Furthermore, multiple sequence alignment revealed that the NRP protein, particularly its DCD domain, is highly conserved across diverse plant species, including *Arabidopsis thaliana*, soybean, and tobacco (Fig. S3). Notably, the key NRP residues predicted to mediate PevD1 binding are conserved among these species. These observations suggest that PevD1 may recognize NRP through a conserved molecular mechanism across different host plants.

### PevD1 exhibits higher binding affinity with NRP

**Figure 2:**
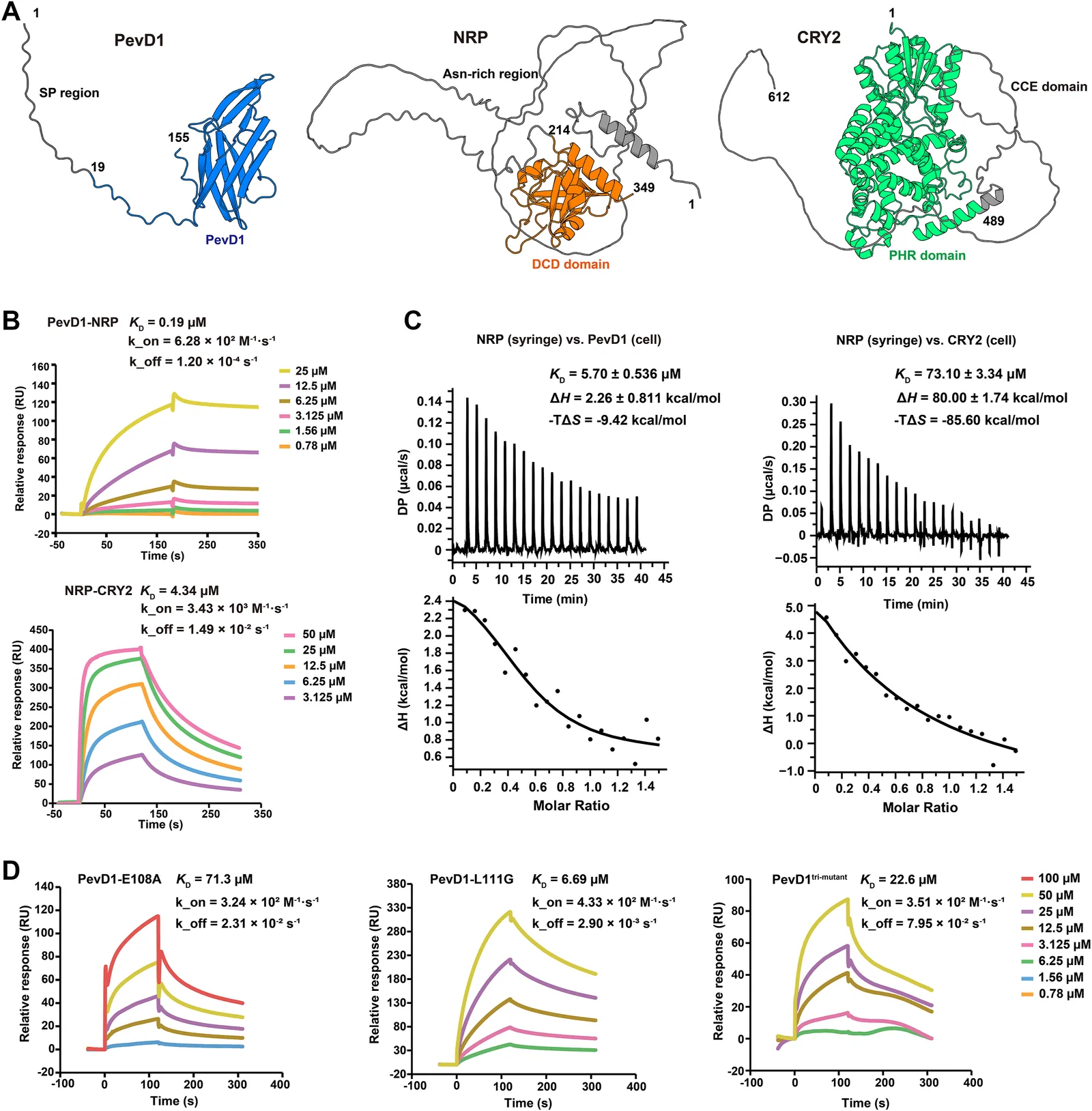
**Binding affinity analysis of PevD1 and CRY2 with NRP.***A,* cartoon representation of sequences and domains of PevD1, NRP, and CRY2. *B,* SPR analysis shows the interaction between PevD1, CRY2 and NRP, performed using the Biacore 8K system with the corresponding sensorgrams is displayed. *C,* Isothermal titration calorimetry binding analyses of PevD1 and CRY2 with NRP, respectively. *D,* analysis of the binding affinity of PevD1 mutants with NRP using SPR. Sensorgrams show the binding responses of different PevD1 mutants with NRP at a series of concentrations. NRP, asparagine-rich protein; CRY2, cryptochrome 2; SPR, surface plasmon resonance.

To elucidate the interaction mechanisms between PevD1, CRY2 and NRP, we first expressed and purified recombinant PevD1, the PHR domain of CRY2, and the DCD domain of NRP in *Escherichia coli* (Figs. 2*A*, S4). The binding affinities between these proteins were then quantitatively assessed using surface plasmon resonance (SPR). SPR analysis revealed a strong interaction between PevD1 and NRP, with a dissociation constant (*K*_D_) of 0.19 μM (Fig. 2*B*). In contrast, the binding affinity between NRP and the CRY2 was significantly weaker, exhibiting a *K*_D_ value of 4.34 μM (Table S2). To further validate these findings, we conducted isothermal titration calorimetry (ITC) experiments (Fig. 2*C*). Consistent with the SPR results, ITC confirmed that PevD1 binds more strongly to NRP (*K*_D_ = 5.7 μM) than that of CRY2 (*K*_D_ = 73.1 μM). The positive ΔH of ITC indicates that the binding is entropy-driven, primarily arising from hydrophobic interactions and desolvation effects. To quantitatively characterize the binding kinetics between PevD1 and NRP, biolayer interferometry was performed (Fig. S5). Kinetic fitting yielded a dissociation constant (*K*_D_) of 5.7 μM with a high correlation coefficient (R^2^ = 0.9961). A strong linear correlation was observed between the response values and protein concentrations, indicating high data reliability. Consistent *K*_D_ values ranging from 3.4 to 7.2 μM were obtained from three independent replicates, suggesting that PevD1 binds NRP with stable and high affinity.

Through structural prediction and analysis of the PevD1-NRP complex, we identified multiple stable interaction pairs at the binding interface, which may play a crucial role in their binding. To validate the predicted structural model and elucidate the precise interaction mechanism between PevD1 and NRP, we performed site-directed mutagenesis on four key interfacial residues of PevD1: D31, E108, L111, and D150. First, single-point mutations were introduced at residues E108 (hydrogen-bonding residue) and L111 (hydrophobic interaction residue) of PevD1 to alanine and glycine, respectively, resulting in PevD1^E108A^ and PevD1^L111G^. Compared with WT PevD1 (*K*_D_ = 0.19 μM), both PevD1^E108A^ and PevD1^L111G^ showed markedly reduced binding to NRP, with *K*_D_ values of 71.3 μM and 6.69 μM, respectively, as determined by SPR (Fig. 2*D*). These values correspond to approximately 374-fold and 35-fold decreases in affinity relative to WT PevD1, indicating that E108 makes a major contribution to NRP recognition. To further assess the combined contribution of additional predicted interfacial residues, we generated a triple mutant, PevD1^tri-mutant^ (D31A/D150A/L111G), in which D31 and D150, predicted to participate in salt-bridge interactions, were substituted with alanine, while L111 was substituted with glycine. SPR analysis demonstrated that PevD1^tri-mutant^ also exhibited a substantial reduction in affinity for NRP, with a *K*_D_ value of 22.6 μM (Fig. 2*D*), further weakening the binding compared to the Leu111 single mutant. These findings confirm that D31, E108, L111, and D150 are essential for the PevD1-NRP interaction, corroborating the accuracy of our structural predictions. Additionally, the identification of these key residues provides a molecular foundation for the rational design of PevD1-targeted inhibitors to disrupt PevD1-NRP interaction.

### Crystallographic analysis of PevD1

Despite our prior computational analysis of the PevD1-NRP interaction pattern and subsequent MD simulations based on predicted structures, the precise molecular mechanisms governing the interaction between PevD1 and NRP remain unresolved due to insufficient structural insights. To elucidate this, we first pursued the crystallization of the DCD domain of Arabidopsis NRP using X-ray diffraction. To enhance protein solubility, we conducted extensive optimization of expression conditions. Ultimately, fusion of NRP with a maltose-binding protein (MBP) tag enabled the production of a stable, homogeneous, and monomeric MBP-NRP construct. However, crystallographic analysis revealed that only the MBP domain generated interpretable electron density, while the NRP region remained undetectable (Fig. S6).

In parallel, through systematic screening and optimization, we successfully obtained a well-diffracting single crystal of PevD1 (Fig. S7*A*), which displayed an elongated cuboidal morphology. The high-resolution crystal structure of PevD1 was determined at 1.35 Å resolutions, providing detailed structural insights into the protein. Compared with the previously reported PevD1 crystal structure resolved at 1.54 Å resolution (27), the present structure Protein Data Bank (PDB ID: 9LDN) offers enhanced structural detail while retaining a highly similar overall conformation, with a backbone RMSD of 0.076 Å between the two structures. However, notable conformational variations were observed in the loop region encompassing residues Q138-P140 (Fig. S7*B*), for which complete electron density was resolved. Importantly, this region lies outside the PevD1-NRP binding interface and does not make direct contacts with NRP. Consequently, these local conformational differences are unlikely to influence the protein–protein interaction. Instead, the observed structural variations may reflect differences in crystallization conditions. Despite efforts to elucidate the crystal structure of the PevD1-NRP complex, we were unable to obtain a resolvable structure. Nevertheless, the high-resolution PevD1 structure serves as a valuable foundation for the rational design of targeted inhibitors blocking PevD1-NRP interactions.

### Discovery of compound A6 as a PevD1-NRP interaction blocker

**Figure 3:**
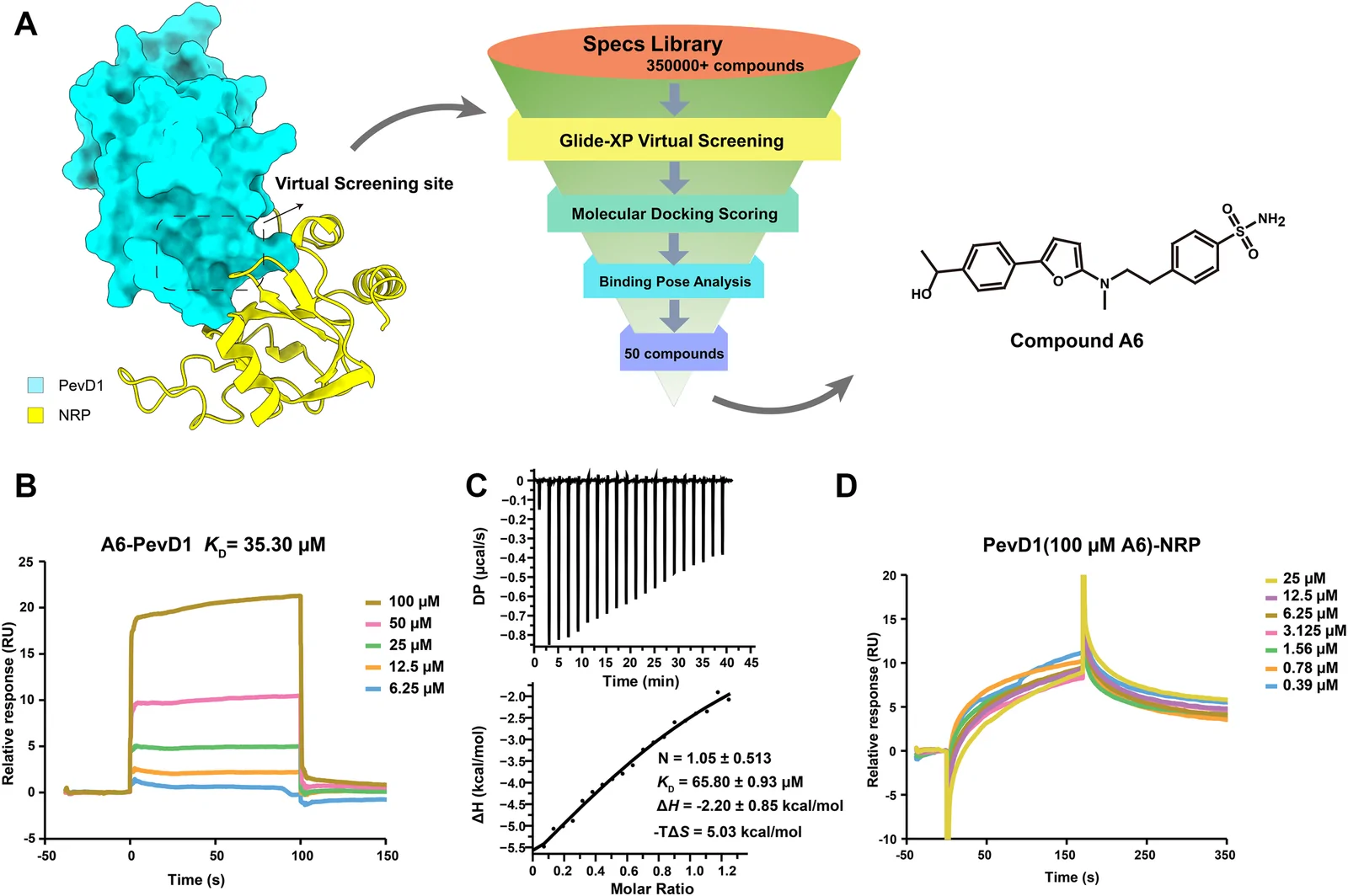
**Compound A6 demonstrates potent inhibitory activity against the PevD1-NRP interaction.***A,* virtual screening was conducted based on the interaction interface of PevD1 and NRP. The middle panel displays the flowchart of the virtual screening process, while the right panel shows the structural formula of the hit compound A6. *B,* SPR analysis of the binding affinity of A6 against PevD1. *C,* isothermal titration calorimetry analysis of A6-PevD1 interaction. *D,* the SPR results demonstrate the blocking effect of 100 μM compound A6 on the PevD1-NRP interaction. NRP, asparagine-rich protein; SPR, surface plasmon resonance.

Following *in silico* and biophysical analysis that together helped identify putative interfacial residues that contribute to the interaction of PevD1 and NRP, we sought to identify specific PPI inhibitors capable of disrupting this complex, thereby attenuating the detrimental effects of fungal infection on Arabidopsis and other plant species. A structure-based virtual screening approach was employed targeting the PevD1-NRP interaction, a highly conserved endogenous plant receptor. To preserve NRP-mediated physiological signaling pathways, our inhibitory strategy was specifically designed to selectively antagonize PevD1, thereby minimizing off-target effects. Structural analysis of the putative PevD1-NRP interaction identified a potential druggable pocket on PevD1 at the binding interface (Fig. 3*A*). This site was designated as the docking target for virtual screening using the Glide module (Schrödinger software suite; https://www.schrodinger.com/) in extra precision (XP) mode. Total number of 271,089 compounds form the Specs commercial compound library (https://www.specs.net/) was systematically screened, and candidate compounds were selected based on docking scores and visual inspection. After rigorous evaluation, 50 top-ranking compounds (Table S4) demonstrating optimal binding conformations and favorable Glide scores were prioritized for subsequent biological activity validation (Fig. 3*A*).

**Figure 4:**
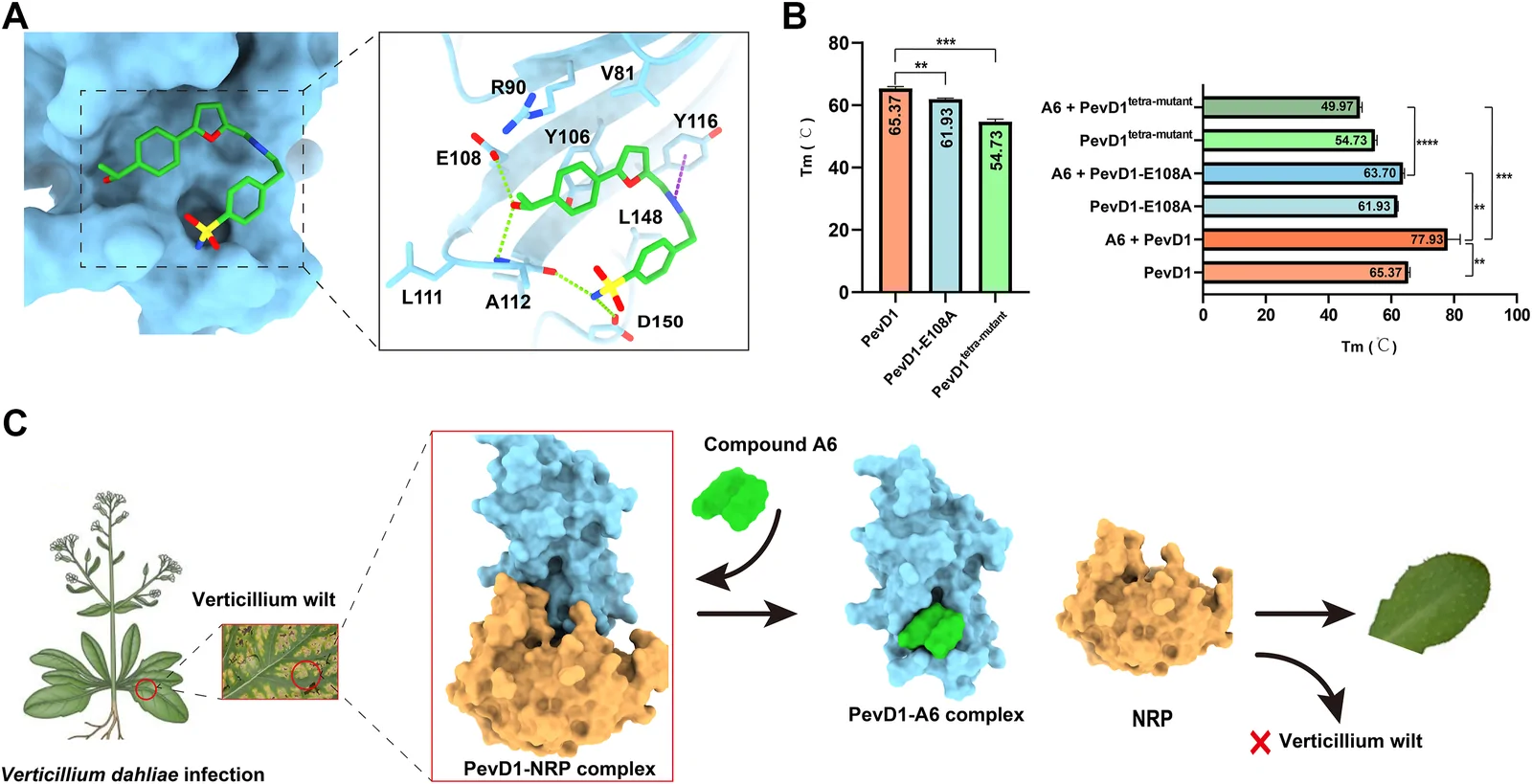
**Prediction and mechanistic validation of compound A6's mode of action.***A,* predicted binding mode and detailed interaction pattern of compound A6 in the PevD1. Key residues for the binding of A6 are shown as *cyan stick*. *B,* the DSF results demonstrate the thermal stability of different PevD1 proteins and the effect of compound A6 on protein stability. Data are presented as mean ± standard deviation (n = 3). ∗*p* < 0.05, ∗∗*p* < 0.01, ∗∗∗*p* < 0.001, ∗∗∗∗*p* < 0.0001 (*t* test). *C,* the mode of action diagram of compound A6 in exerting antiverticillium wilt effects by blocking the PevD1-NRP interaction. NRP, asparagine-rich protein.

The binding affinities of 50 candidate compounds to PevD1 were assessed using SPR. Among these, compound A6 (Fig. 3*A*) demonstrated measurable binding to PevD1, exhibiting a *K*_D_ value of 35.3 μM (Fig. 3*B*, Table S2). To further elucidate the interaction between A6 and PevD1, ITC experiment was employed. The ITC results corroborated the SPR findings, confirming a *K*_D_ value of 65.8 μM (Fig. 3*C*), thereby validating the binding interaction. To determine whether A6 disrupts the PevD1-NRP interaction, SPR-based competitive binding assays were conducted. As illustrated in Figure 4*D*, at a fixed concentration gradient of PevD1, the presence of 100 μM A6 significantly attenuated the binding signal between PevD1 and NRP compared to the control (Fig. 2*B*). This observation indicates that A6 effectively inhibits the PevD1-NRP interaction. Collectively, these results demonstrate that A6 binds to PevD1 in a dose-dependent manner and competitively inhibits its association with NRP.

### Mechanistic analysis of compound A6 binding to PevD1

The interaction details of the active compound A6, obtained through molecular docking simulations, were further analyzed. In these predictions, A6 exhibited a complementary fit within the pocket. For detailed interaction, the hydroxyl group at one end of the A6 establishes hydrogen bonds with residue A112, E108, while the central nitrogen atom can exist in a protonated state at neutral pH, resulting in a positively charged amine species, as supported by protonation state prediction and form cation-π interaction with residue Y116. At the opposite end, the sulfonyl group forms hydrogen bonds with the side chain of residue D150 and the main chain of residue A112. These interactions stabilize A6 binding at the binding site (Fig. 4*A*).

To elucidate the binding interaction between compound A6 and the protein PevD1, we conducted site-directed mutagenesis targeting four critical interfacial residues of PevD1 (R90, Y106, E108, and D150). Based on docking analysis of the PevD1 tetra-mutant and single mutants with A6, the tetra-mutant and E108A mutant were selected as they most substantially affected both docking scores and interaction networks. The binding site of A6 on PevD1 was subsequently evaluated using nanoDSF (nano Differential Scanning Fluorimetry) analysis. As illustrated in Figure 4*B*, the WT PevD1 exhibited a melting temperature (*T*_m_) of 65.4 °C. In contrast, the PevD1-E108A mutant displayed a reduced *T*_m_ value of 62 °C, while the PevD1^tetra-mutant^ (R90A/Y106A/E108A/D150A) demonstrated a more pronounced destabilization, with a *T*_m_ value of 54.7 °C. To further assess the impact of compound A6 on protein thermal stability, we analyzed its effect on the *T*_m_ values of the tested variants. Upon binding of A6, the WT PevD1 exhibited a significant increase in thermal stability, with its *T*_m_ rising to 78 °C (Δ*T*_m_ = +10.7 °C). The significant positive thermal shift observed strongly suggests that compound A6 engages in direct binding with PevD1, likely conferring structural stabilization. In contrast, the PevD1-E108A mutant displayed only a marginal thermal shift upon A6 binding (Δ*T*_m_ = 1.7 °C), indicating that the E108A mutation substantially diminished the binding affinity between A6 and PevD1. Notably, the PevD1^tetra-mutant^ exhibited a complete loss of A6-induced thermal stabilization (Δ*T*_m_ = −4.7 °C), demonstrating that A6 specifically targets PevD1 *via* a binding site critically dependent on these four key residues.

In summary, compound A6 exhibits inhibitory potential by stably binding to PevD1 and disrupting its interaction with NRP and thus resistance to Verticillium wilt caused by *Verticillium dahliae* infection in plants (Fig. 4*C*). Experimental data confirm that A6 competitively occupies the PevD1 binding site, effectively blocking the PevD1-NRP complex formation. Notably, the A6-binding region on PevD1 is also involved in interactions with other plant proteins, implying broader functional interference with multiple pathogenicity-related pathways. These findings highlight the therapeutic promise of developing PPI inhibitors targeting PevD1. This study offers critical structural insights and a robust theoretical foundation for designing innovative antifungal agents directed against PevD1-mediated virulence mechanisms.

## Discussion

This study elucidates the molecular determinants underlying the interaction between the *Verticillium dahliae* effector protein PevD1 and NRP, providing a detailed analysis of its molecular mechanisms and potential applications for therapeutic development. We systematically elucidate the molecular mechanism underlying the interaction between the *Verticillium dahliae* effector protein PevD1 and the *Arabidopsis* NRP protein. SPR binding assays show that PevD1 specifically binds to the DCD domain of NRP (*K*_D_ = 0.19 μM), with a binding affinity approximately 23 times higher than that of CRY2 (4.34 μM), thereby confirming the competitive interaction between PevD1 and CRY2 for binding to NRP. Nevertheless, we acknowledge that intrinsically disordered regions can contribute to protein interactions through transient or context-dependent contacts. Therefore, we cannot completely rule out the possibility that full-length NRP may exhibit different, potentially higher, affinity for CRY2 than the isolated DCD domain.

Then, we use AI model AF-multimer to predict the interaction pattern of NRP-PevD1 and NRP-CRY2. Then MD was conducted to further predict residues that contribute to the stability of protein complex. With the results of prediction, E108 and L111 were tested as individual single-point mutants (E108A and L111G), while D31 and D150 were not analyzed as individual single mutants but were instead incorporated into a triple mutant (D31A/D150A/L111G) to assess their collective contribution to binding stability. SPR analysis demonstrated that the E108A mutation caused the most pronounced reduction in binding affinity, indicating that E108 is the primary determinant of the PevD1-NRP interaction. In contrast, mutations involving L111, D31, and D150 resulted in more moderate decreases in affinity, suggesting supportive but secondary roles in stabilizing the interface.

Despite extensive crystallization attempts, we were unable to resolve the PevD1-NRP complex structure and other structural methods like HDX-MS, or NMR, will be required in future work to define the interface at atomic resolution. Structural analysis also provided the high-resolution crystal structure of PevD1, revealing conformational changes in the Q138-P140 region (Fig. S7*B*). However, this region is located outside the PevD1-NRP binding interface and does not make direct contacts with NRP. Therefore, these local conformational differences are unlikely to affect the PPI.

In the PevD1-NRP-CRY2 regulatory module, the pathogen effector PevD1 acts upstream by competitively binding to NRP, thereby disrupting the NRP-CRY2 interaction and reprogramming host developmental signaling, including CRY2 localization and flowering responses. Compared with targeting the host susceptibility factor NRP, focusing on the pathogen effector PevD1 represents a more advantageous strategy. Modulating NRP may impair its intrinsic physiological functions and lead to pleiotropic effects, as host proteins are involved in multiple regulatory pathways. In contrast, pathogen-derived effectors offer higher specificity; PevD1 is absent from the host genome, enabling selective intervention with minimal off-target effects while preserving normal CRY2-mediated photoperiod regulation. Importantly, our sequence alignment and structural comparison revealed that the DCD domain of NRP, including the key residues involved in binding PevD1 and CRY2, is highly conserved among Arabidopsis, tobacco, soybean, and rice, and the overall structures of these NRP homologs are also highly similar (Fig. S3). These findings suggest that the PevD1–NRP interaction interface is evolutionarily conserved across diverse plant species, supporting the possibility that disrupting this interaction may provide a broadly applicable strategy for enhancing disease resistance in multiple crops.

To further inhibit the PevD1-NRP interaction, we performed structure-based virtual screening and identified A6 as a small-molecule disruptor that specifically binds PevD1 and effectively blocks its interaction with NRP. Notably, PevD1 also targets the senescence regulator ORE1 in both *Arabidopsis* and cotton, promoting leaf senescence (15). Although the predicted ORE1-binding region differs from the NRP-binding interface, we observed partial spatial overlap between the ORE1-associated surface and the predicted binding site of compound A6 on PevD1. Together, these findings highlight that targeting PevD1 can prevent effector-mediated reprogramming of host signaling without disrupting endogenous protein functions, thereby enhancing intervention selectivity and safety. This supports a “molecular protector” strategy, in which targeting pathogen effectors may provide superior outcomes compared with direct manipulation of host factors.

In summary, this study elucidates the molecular determinants underlying the interaction between the *Verticillium dahliae* effector protein PevD1 and NRP in Arabidopsis, providing insights into both its molecular mechanisms and structural characteristics. However, the present study is primarily based on *in vitro* biochemical, biophysical, and computational analyses, and therefore does not directly establish the biological significance of the PevD1-NRP interaction during plant infection. Future in planta studies will be required to validate the functional relevance of this interaction in the context of *V. dahliae* pathogenesis. Additionally, we investigate potential strategies for designing small-molecule inhibitors targeting PevD1. Future research should focus on further exploring the interaction patterns between PevD1 and host plant proteins, which may clarify the molecular basis of *Verticillium dahliae*'s infection process. Moreover, the novel PPI inhibitor of PevD1 identified in this study could undergo further optimization to enhance its binding affinity. However, the biological efficacy of compound A6 in plants remains to be established. Its ability to suppress *Verticillium dahliae*–induced early flowering or broader pathogen-associated developmental reprogramming has not yet been tested, nor has its potential impact on fungal virulence been evaluated. Future studies should therefore validate A6 within plant-pathogen systems, including assessments of flowering phenotypes, CRY2 subcellular localization, and the expression of downstream regulators such as CO, FT, and SOC1(16). Rapid in planta assays, such as transient expression in *Nicotiana benthamiana*, could be used to determine whether A6 disrupts the PevD1–NRP interaction and alters CRY2 localization. In parallel, infection assays with *V. dahliae* are needed to assess potential anti-virulence effects. Genetic materials with altered expression of PevD1, NRP, or CRY2(16) could further clarify whether A6 restores NRP-mediated regulation and reverses PevD1-induced phenotypes. Overall, these studies are essential to determine whether targeting the PevD1-NRP interface is sufficient to counteract effector-driven host reprogramming and to evaluate the translational potential of A6 as a selective anti-virulence agent.

## Experimental procedures

### Protein complex structure prediction

AF-Multimer is an advanced protein structure prediction tool derived from AlphaFold2, specifically optimized for modeling the three-dimensional structures of protein complexes (28). In this study, we first input the full-length protein sequences of CRY2 and NRP, as well as PevD1 and NRP, into AlphaFold-Multimer. This allowed us to predict the three-dimensional structures of both protein complexes, along with their corresponding PAE maps, which assess the confidence of inter-residue distance predictions. Subsequently, to model the ordered regions of these complexes, we extracted the sequences corresponding to the structured domains of CRY2 (residues 4–495) and NRP (residues 216–349), as well as PevD1 (residues 31–152) and NRP, and submitted them to AF-multimer. For each pair of complex, five models are generated; we showed the confidence of each model (Fig. S2), and choose model two of CRY2-NRP complex prediction with a highest confidence 0.566 and model five of PevD1-NRP complex with a highest confidence 0.662. This step generated the predicted structures for the ordered regions of both complexes.

### MD simulation analysis

The Schrödinger software suite is a comprehensive computational biology platform designed to perform a broad range of computational tasks. Among its modules, Desmond enables MD simulations of protein complexes (29). In this study, the Desmond module was used to construct MD systems, employing the OPLS4 force field (30) to perform three parallel MD simulations for each model. The initial complex structures were placed in a cubic simulation box and solvated using the TIP3P water model. Sodium and chloride ions were added to neutralize the system and maintain physiological pH. All MD simulations were performed under the NPT ensemble, with the temperature and pressure maintained at 300 K and 1 bar, respectively. For each system, three independent parallel MD simulations of 200 ns were carried out with a time step of 2 fs. Trajectories were recorded every 2 ps, resulting in a total of 1000 frames for subsequent analysis.

To investigate the dynamic interactions between the two proteins, we employed the *analyze_trajectory_ppi.py* script (Schrödinger) to perform a statistical assessment of residue-residue interactions across the MD trajectories. The analysis was conducted using three independent parallel trajectories for each protein complex. Interactions exhibiting a persistence of >70% across all trajectory frames were identified and classified as stable PPIs. We employed the *gmx sham* tool to construct free energy landscape (FEL) maps from the MD simulation trajectories of the CRY2-NRP and PevD1-NRP complexes. Three independent trajectories for each protein complex were computed for the conformational FEL, focusing on the PPI interface. The resulting FEL maps were subsequently analyzed to identify low-energy conformational states.

### Molecular docking

The Glide module within the Schrödinger software suite was used to perform two rounds of molecular docking and virtual screening as part of the PevD1 inhibitor identification process. For the initial screening, the advanced mode in Glide was applied to identify potential inhibitory compounds from the SPECS commercial compound library. In the second round of screening, both shape screening and advanced mode was employed to further refine the selection process.

### Construction of protein expression vectors

PevD1 (effector protein, Gene ID: 20732519) from *Verticillium dahliae*, CRY2 (cryptochrome 2, Gene ID: 839529) from *A. thaliana*, and NRP (DCD domain protein, Gene ID: 834,210) from *A. thaliana* were used in this study. The PevD1 gene, excluding the 18-amino acid signal peptide, consists of 468 base pairs encoding 155 amino acids. The NRP^DCD^ gene comprises 408 base pairs encoding 136 amino acids, while the CRY2^PHR^ gene contains 1518 base pairs encoding 506 amino acids. The GST-NRP^DCD^ and CRY2^PHR^ genes were cloned into the pET-15b expression vector, whereas the PevD1 and MBP-NRP^DCD^ genes were cloned into the pET-28a expression vector. All vectors were designed with an N-terminal 6×His affinity tag and a thrombin-specific cleavage site (LVPR↓GS) to facilitate the removal of the affinity tag after recombinant protein purification.

Using the constructed pET-28a-PevD1 plasmid as a template, site-directed mutagenesis was performed to generate single, triple, and quadruple mutants. Mutation-specific primers containing the desired nucleotide substitutions were designed according to the targeted sites (Table S1). PCR reactions were carried out following the instructions provided with the Nanjing Vazyme kit. After amplification, the parental plasmid was digested with DpnI endonuclease, and the treated products were directly transformed into *E. coli* DH5α competent cells. Plasmids were then extracted and verified by DNA sequencing to confirm the successful introduction of the intended mutations.

### Protein expression and purification

The Shuffle T7 *E. coli* competent cells carrying the target plasmid (Shanghai Weidi Biotechnology) were inoculated into Luria-Bertani medium and cultured at 37 °C with shaking until the optical density at 600 nm reached 1.0. The culture was then cooled to 16 °C, and isopropyl-β-D-thiogalactopyranoside was added to a final concentration of 0.3 mM for low-temperature induction (16 °C for 18 h). After cell harvest, the pellet was resuspended in lysis buffer (25 mM Tris, 300 mM NaCl, 20 mM imidazole, pH 8.0), and a nuclease and protease inhibitor cocktail (TargetMol) was added. Cells were then lysed using a high-pressure cell disruptor, and the cell debris was removed by centrifugation at 4 °C. For nickel affinity chromatography (Ni Sepharose FF, GE Healthcare), recombinant proteins were captured using a gravity-flow column and eluted using a high-imidazole buffer (25 mM Tris, 300 mM NaCl, 300 mM imidazole, pH 8.0). For recombinant proteins with a GST tag, glutathione agarose beads (Smart-Lifesciences) were used for affinity chromatography, and the elution buffer consisted of Tris-HCl solution containing 10 mM reduced glutathione (50 mM Tris, 300 mM NaCl, pH 8.0). After concentration with a centrifugal ultrafiltration tube (10 kDa molecular weight cutoff, MCE), the eluate was further purified by gel filtration chromatography using a pre-packed HiLoad 16/600 Superdex 200 pg column (Cytiva), with 25 mM Tris and 200 mM NaCl (pH 8.0) as the elution buffer. The final purified protein was concentrated to 10 mg/ml, aliquoted, and stored at −80 °C. Protein concentration was determined using a NanoDrop spectrophotometer (Thermo Fisher Scientific) at 280 nm, with the actual concentration calculated based on the theoretical molar extinction coefficient.

### SPR analysis

Based on the pre-enrichment experiment, the optimal coupling conditions were determined, with recombinant GST-NRP protein showing the strongest electrostatic adsorption in 10 mM sodium acetate buffer at pH 4.0. This condition was selected for immobilization. The protein was diluted to 10 μg/ml and immobilized onto S-series CM5 sensor chips (Cytiva) using the standard amine coupling method, achieving a final response value (RU) of 1000 ± 50. For ligand interaction analysis, PevD1 and CRY2 proteins were desalted and exchanged into 1 × HBS-EP running buffer (10 mM Hepes, 150 mM NaCl, 3 mM EDTA, 0.025% (v/v) Tween-20, pH 7.4), with gradient concentrations (3.125–100 μM) prepared. The analysis parameters were set as follows: association time of 120 s, dissociation time of 180 s, flow rate of 30 μl/min, and surface regeneration after each cycle with 10 mM glycine-HCl (pH 1.5).

To evaluate the binding properties of small molecule compounds, recombinant PevD1 protein was diluted to 20 μg/ml in pH 4.0 sodium acetate buffer and immobilized onto the CM5 chip, achieving a binding response of 6000 ± 200 RU. The compound stock solution (10 mM in 100% DMSO) was serially diluted in HBS-EP buffer to final concentrations of 3.125 to 100 μM, with 5% DMSO. The experiment was conducted using dynamic association mode with an association time of 100 s, dissociation time of 120 s, and 50% DMSO washing after each cycle. The DMSO concentration ranged from 4.5% to 5.8%, and solvent corrections were applied every 48 cycles. Raw data were processed using Biacore 8K (https://www.cytivalifesciences.com.cn/zh/cn/products/items/biacore-8-series-spr-systems-p-56116?psmenu=2) evaluation software for simplification, dual referencing, and solvent correction. The *K*_D_ values for each compound were calculated using the steady-state affinity model with a constant Rmax.

### ITC

All thermodynamic measurements were conducted using a microcalorimeter with heat-pump calibration (MicroCal PEAQ-ITC, Malvern Panalytical). The experiments were performed at a constant temperature of 25 °C under buffer-matching conditions (equilibrium buffer: 20 mM Tris-HCl, 200 mM NaCl, pH 8.0), with the sample cell stirred at 750 rpm. For PPI measurements, both the receptor and ligand proteins were dialyzed into the same buffer system to minimize solvent heat effects. The receptor protein was diluted to a final concentration of 40 μM in ITC buffer, and 500 μl was injected into the sample cell. The ligand protein was prepared by diluting a high-concentration stock solution to 300 μM in the same buffer system and loaded into the syringe (200 μl volume). The titration procedure followed a stepwise mode: an initial coarse injection of 0.4 μl was used to balance the baseline signal, followed by 19 precise injections (2 μl each). Injection intervals were set to 4 s for initial equilibrium and 120 s for steady-state data collection.

For protein-compound interaction measurements, the compound stock solution (50 mM in DMSO) was serially diluted in ITC buffer to 500 μM, while the target protein was diluted to 80 μM. The final DMSO concentration in both samples was maintained at 1% to eliminate solvent interference. Raw heat flow data were processed using PEAQ-ITC (https://www.malvernpanalytical.com.cn/products) analysis software (Malvern) for baseline drift correction, curve fitting, and noise filtering. Binding model selection was based on the minimum χ^2^ principle, and the one-site binding model was applied to calculate the binding constant (*K*_D_), stoichiometry (n), enthalpy change (Δ*H*), and entropy change (Δ*S*). Each experiment was performed in triplicate, with the software iteration convergence threshold set to 1e-6. Residual plots were used to assess the goodness of fit.

### Bio-layer interferometry

Recombinant MBP-NRP protein was incubated with a tenfold molar excess of D-biotin (Yeasen) at 4 °C for 4 h. The mixture was then exchanged into desalting buffer (10 mM Hepes, 150 mM NaCl, 3 mM EDTA, 0.025% v/v Tween-20, pH 7.4) using a desalting column to remove free biotin. The biotinylated MBP-NRP protein was diluted to a final concentration of 25 μg/ml and coupled to streptavidin (SA) biosensor probes in the Octet RED96e system (Sartorius) *via* the ligand immobilization procedure. The contact time was set to 600 s to ensure stable coupling, with the response reaching 0.35 nm, indicating a non-mass transfer adsorption level. All molecular interaction experiments were performed at a constant temperature of 25 °C with shaking at 1000 rpm in analysis buffer (10 mM Hepes, 150 mM NaCl, 3 mM EDTA, 0.025% Tween-20, 0.5% BSA, 0.05% DMSO, pH 7.4). The experimental design utilized a four -channel comparative mode: Probes 1 to 2 were blank controls (buffer only), Probes 3 to 4 contained gradient concentrations of PevD1 protein (20, 10, 5, 2.5, 1 μM). Each detection cycle consisted of three steps: baseline equilibrium (60 s), association phase (300 s), and dissociation phase (600 s). The biosensor surface was regenerated using 10 mM glycine-HCl (pH 1.5). Raw interference signals were processed using Data Analysis HT software (v11.0, Sartorius; https://facilities.bioc.cam.ac.uk/files/media/data_analysis_octet.pdf) through three steps: baseline correction (subtracting buffer reference signals from probes 1–2), kinetic fitting (using a 1:1 binding model (Langmuir) to calculate the association rate constant (k_on_), dissociation rate constant (*k*_off_), and affinity constant (*K*_D_).

### NanoDSF assay

Protein samples (WT or mutant) were diluted in buffer (50 mM Tris, 200 mM NaCl, pH 8.0) to a final concentration of 20 μM. The control group contained 1% (v/v) DMSO, while the experimental group contained 100 μM compound A6 dissolved in 1% DMSO under the same protein concentration. Thermal unfolding experiments were performed using a NanoDSF instrument Prometheus NT.48. The temperature gradient was set from 20 °C to 95 °C with a heating rate of 1 °C/min. The intrinsic fluorescence emission ratio at 350 nm/330 nm was continuously monitored as a function of temperature to generate protein melting curves, from which the melting temperature (*T*_m_) was determined. Each sample was measured in triplicate to ensure data reproducibility and reliability. The effect of compound A6 on protein thermal stability was evaluated by comparing the changes in *T*_m_ values (Δ*T*_m_) in the presence and absence of compound A6.

### Protein crystallization and data collection

Initial crystal screening was performed using both sitting-drop vapor diffusion (25 °C) and hanging-drop vapor diffusion (16 °C) methods, employing commercial crystallization screening kits from Hampton Research and Jena Bioscience. For the MBP-NRP protein complex, crystallization conditions were optimized as follows: a 12 mg/ml MBP-NRP protein solution (storage buffer: 10 mM Hepes, 150 mM NaCl, pH 7.5) was mixed in equal volumes (1 μl:1 μl) with crystallization reservoir solution containing 25% (w/v) PEG 1500 and 100 mM MMT buffer (pH 9.0). Precipitant concentration (20–27.5% w/v PEG 1500) and pH (8.6–9.4) were varied in a gradient, with three-dimensional crystals suitable for X-ray diffraction obtained in 20% (w/v) PEG 1500 and 100 mM MMT buffer (pH 8.6).

For apo-PevD1 protein crystallization, the hanging-drop vapor diffusion method was used (16 °C). A 10 mg/ml PevD1 protein solution (20 mM Tris, 150 mM NaCl, pH 8.0) was mixed in equal volumes (1.5 μl:1.5 μl) with a crystallization reservoir solution containing 3.2 M sodium formate. High-quality diffracting crystals were obtained after 28 days. For compound complex crystals, native crystals were soaked in crystallization buffer containing 5 mM small molecule ligand for 6 h. All crystals were cryoprotected in mother liquor containing 20% (v/v) glycerol and flash-frozen in liquid nitrogen. Diffraction data were collected at the Shanghai Synchrotron Radiation Facility (SSRF) BL18U1 beamline (wavelength 0.978 Å). Raw data were processed and integrated using the HKL3000 package (HKL Research, Inc) and XDS software (https://xds.mr.mpg.de/html_doc/XDS.html).

### Structure determination and refinement

After processing with autoPX, the PevD1 structure (PDB ID: 5XMZ) was used as the search model in the Phaser-MR molecular replacement algorithm from the Phenix software (https://phenix-online.org/) suite to solve the phase problem. The PevD1 protein structure was subsequently optimized and refined using Coot and Phenix. Data collection and structure determination statistics for the final solution are provided in Table S3.

### Data availability

The only dataset generated in this study is the protein crystal structure, which has been deposited in the RCSB PDB. The accession ID is 9LDN. All data supporting the findings of this study are available within the manuscript and from the corresponding repository. The original contributions presented in the study are included in the article/supplementary material; further inquiries can be directed to the corresponding author/s.

## Supporting information

This article contains supporting information.

## Conflict of interest

The authors declare that they have no conflicts of interest with the contents of this article.
