# Supplementary material for: Molecular mechanism of fungal effector PevD1-NRP interaction and discovery of a small-molecule disruptor
Source: J Biol Chem. 2026 Aug 10;302(10):113431. doi: 10.1016/j.jbc.2026.113431 (PMC13570309; doi:10.1016/j.jbc.2026.113431)
Supplement: Supplementary file 1 [file mmc1.pdf]

Supporting Information

Molecular Mechanism of Fungal Effector PevD1-NRP Interaction and Discovery of a Small-Molecule Disruptor

Xin Yang[a,b,1], Siyuan Tian[a,1], Chao Xu[a,b], Lu Liu[a], Fang Bai[a,c,*], Xianglei Zhang[a,*]

[a] Shanghai Institute for Advanced Immunochemical Studies and School of Life Science and Technology, ShanghaiTech University, 393 Middle Huaxia Road, Shanghai, China, 201210
[b] Lingang Laboratory, Shanghai, 200031, China.
[c] Shanghai Clinical Research and Trial Center, Shanghai, China, 201210
[1] These authors contributed equally

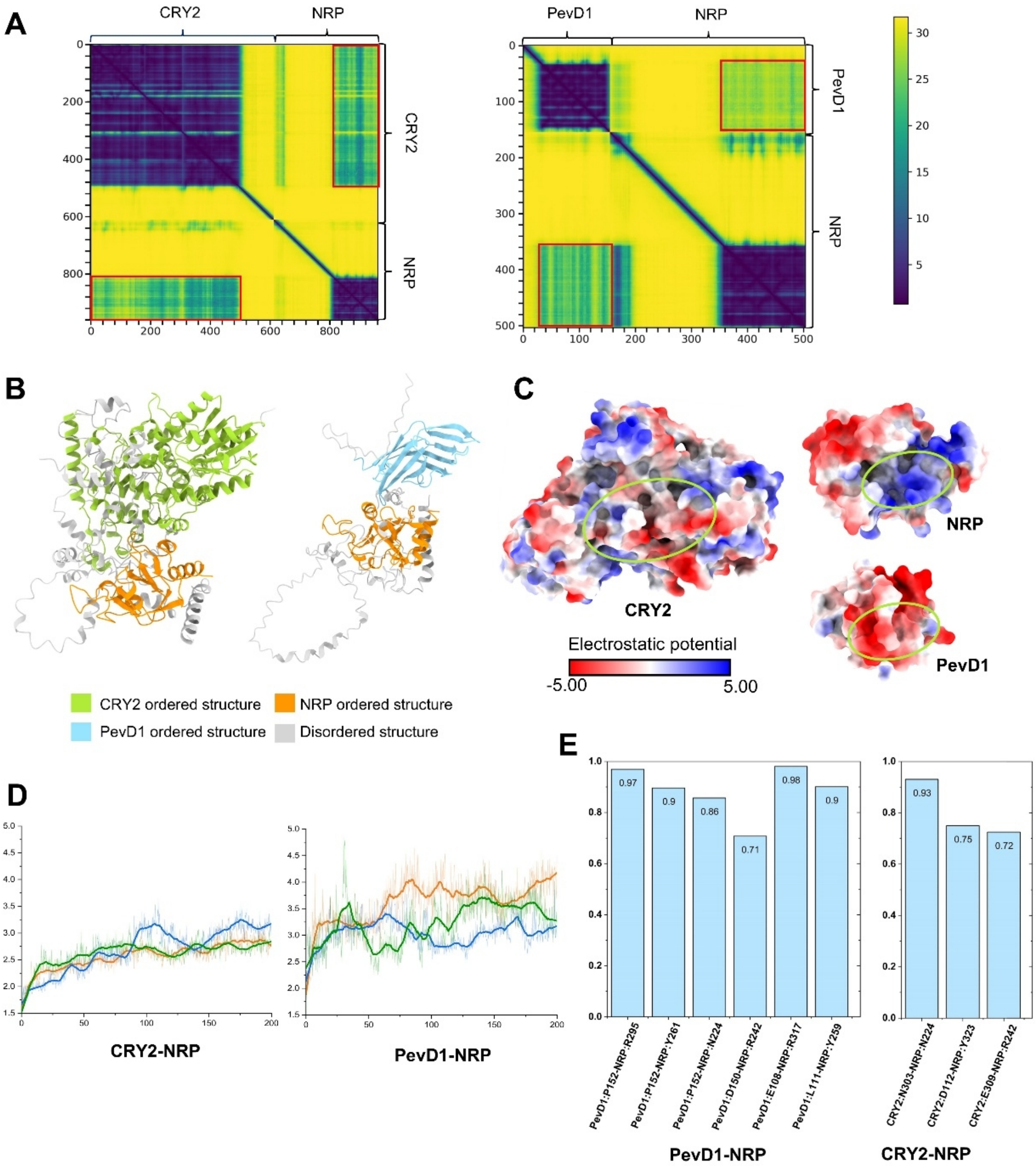

Figure S1. Predicted binding modes of PevD1-NRP and CRY2-NRP complexes. (A) The PAE maps illustrate the dimeric interactions of the CRY2-NRP and PevD1-NRP complexes, as predicted by AF-Multimer. The axes of each map are labeled with the respective protein sequences (CRY2, NRP and PevD1, NRP). Regions highlighted in red indicate potential key interaction sites between the two proteins **(B)** The full-length structural models of the CRY2-NRP and PevD1-NRP complexes were predicted using AlphaFold-Multimer. The ordered domains are defined as follows: CRY2 (residues 4–495, depicted in green), NRP (residues 216–349, depicted in orange), and PevD1 (residues 31–152, depicted in blue). Disordered regions of the proteins are represented in gray. **(C)** The electrostatic potential energy surfaces of CRY2, NRP and PevD1. The green ellipse represents the interaction interface. the electrostatic potential analysis was conducted via APBS electrostatics of Pymol software. **(D)** RMSD of CRY2-NRP and PevD1-NRP complexes across MD trajectories. **(E)** The histogram illustrates the stably interacting residue pairs in the two complexes during the 200ns simulation process.

A

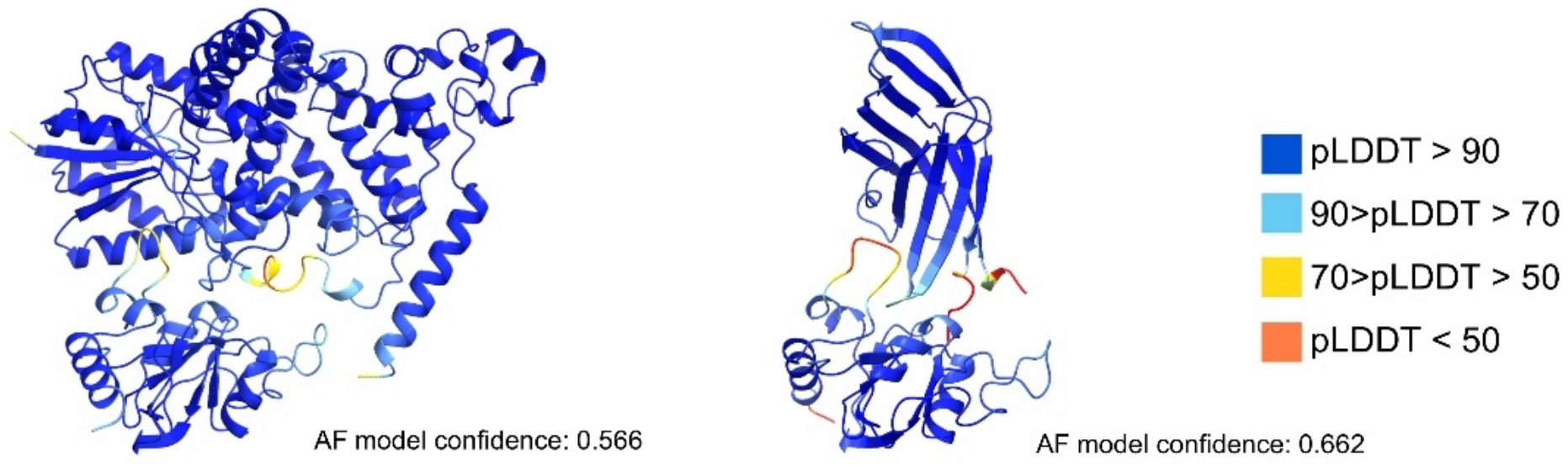

CRY2-NRP predicted complex PevD1-NRP predicted complex

pLDDT distribution on AF-Multimer predicted CRY2-NRP and PevD1-NRP complexes

B

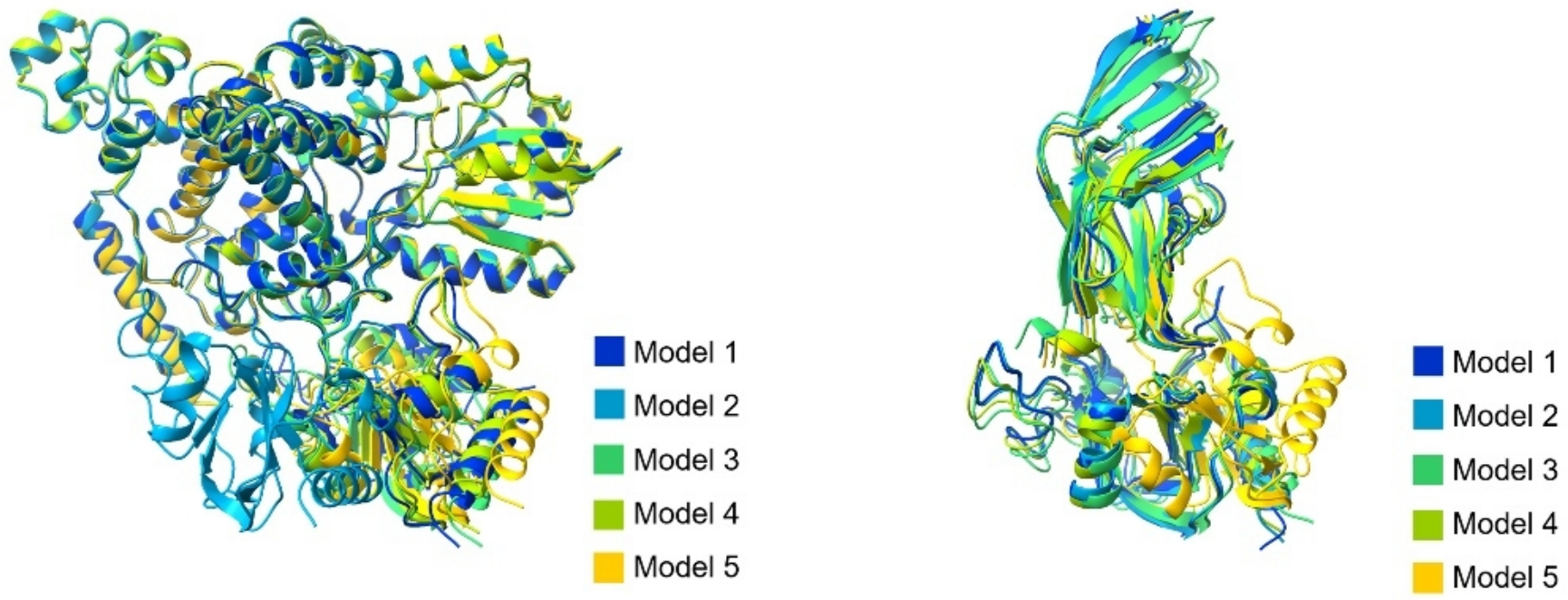

CRY2-NRP predicted complexes overlay PevD1-NRP predicted complexes overlay

Top 5 prediction models overlay of AF-Multimer predicted CRY2-NRP and PevD1-NRP complexes

C

CRY2-NRP complex		PevD1-NRP complex	
Model number	Confidence	Model number	Confidence
Model 1	0.389	Model 1	0.28
Model 2	0.566	Model 2	0.244
Model 3	0.303	Model 3	0.263
Model 4	0.534	Model 4	0.346
Model 5	0.467	Model 5	0.662

Figure S2. Alphafold-multimer predicted complex structures. (A) pLDDT distribution of the top-1 ranked predicted structures for the CRY2-NRP and PevD1-NRP complexes. **(B)** Alignment results of the top-5 predicted complex structures for the two groups. **(C)** Confidence of top-5 AF-multimer predicted PevD1-NRP, CRY2-NRP ordered domain structures.

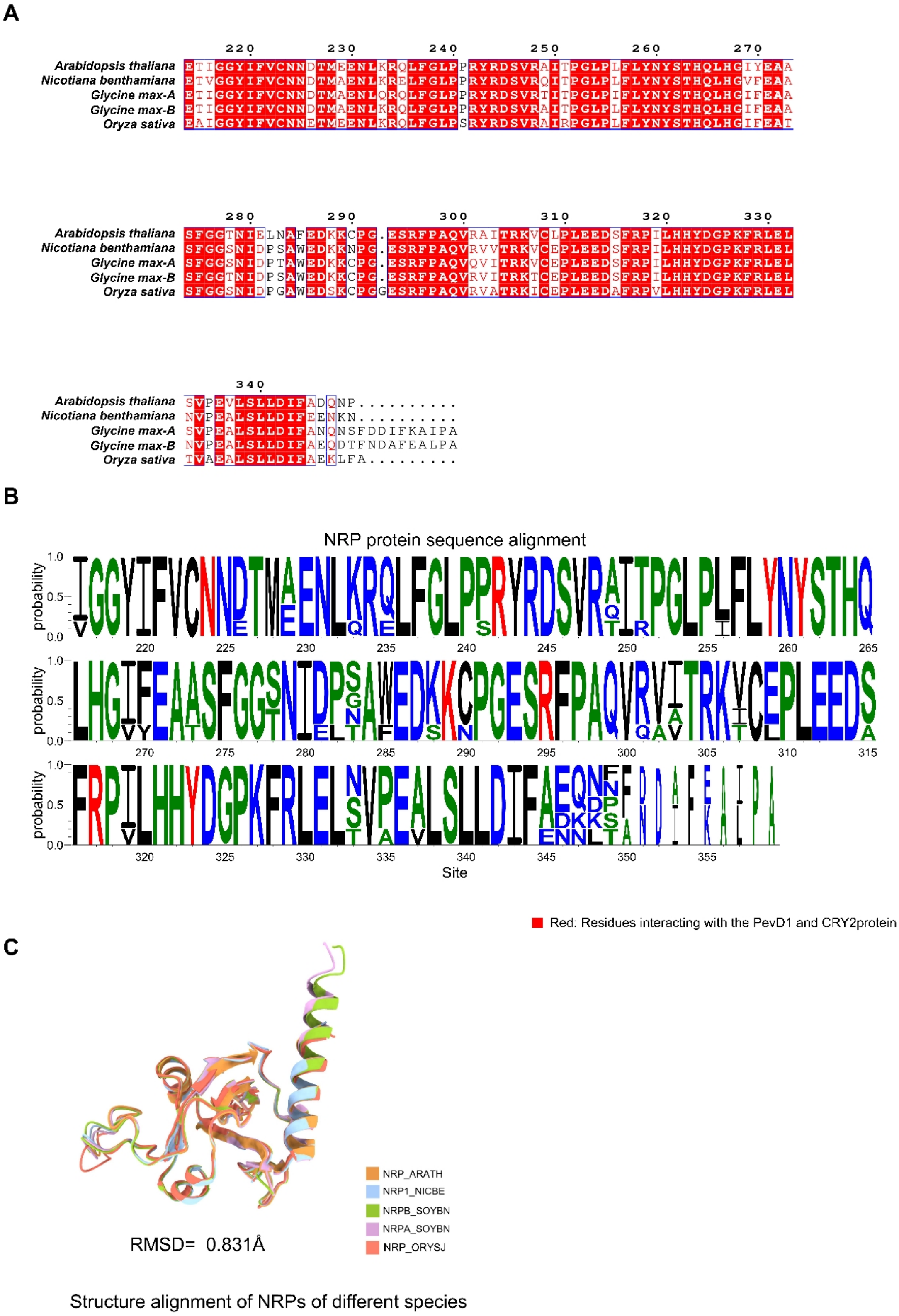

Figure S3. Sequence and ordered-region structural alignment of NRP proteins from different plant species. (A) Multiple sequence alignment of NRP homologs from *Arabidopsis thaliana*, *Nicotiana benthamiana*, *Glycine max* (GmNRP-A and GmNRP-B), and *Oryza sativa*. Based on the interactions between *A. thaliana* NRP and CRY2, as well as between NRP and PevD1, the *A. thaliana* NRP protein was used as the reference sequence for alignment. Highly conserved amino acid residues are

highlighted in red and are predominantly located within the DCD domain of NRP proteins. *Glycine max*-A and *Glycine max*-B represent the two NRP homologs identified in *G. max*, whereas the NRP homolog in *O. sativa* has not yet been assigned a specific name. (B) Conservation analysis of key residues within the DCD domain involved in interactions with CRY2 and PevD1. Amino acid residues identified by molecular dynamics analysis in Figure 1C as critical for binding to either CRY2 or PevD1 are highlighted in red, indicating that these interaction sites are highly conserved among NRP homologs from the examined plant species. (C) Structural superposition of the AlphaFold-predicted NRP proteins from *Arabidopsis thaliana*, *Nicotiana benthamiana*, *Glycine max*, and *Oryza sativa*. The five protein structures exhibited a root-mean-square deviation (RMSD) of 0.831 Å, indicating a high degree of structural conservation among these NRP homologs. Orange, light blue, green, purple, and red represent *A. thaliana* (NRP_ARATH), *N. benthamiana* (NRP1_NICBE), *G. max* (NRPB_SOYBN), *G. max* (NRPA_SOYBN), and *O. sativa* (NRP_ORYSJ), respectively.

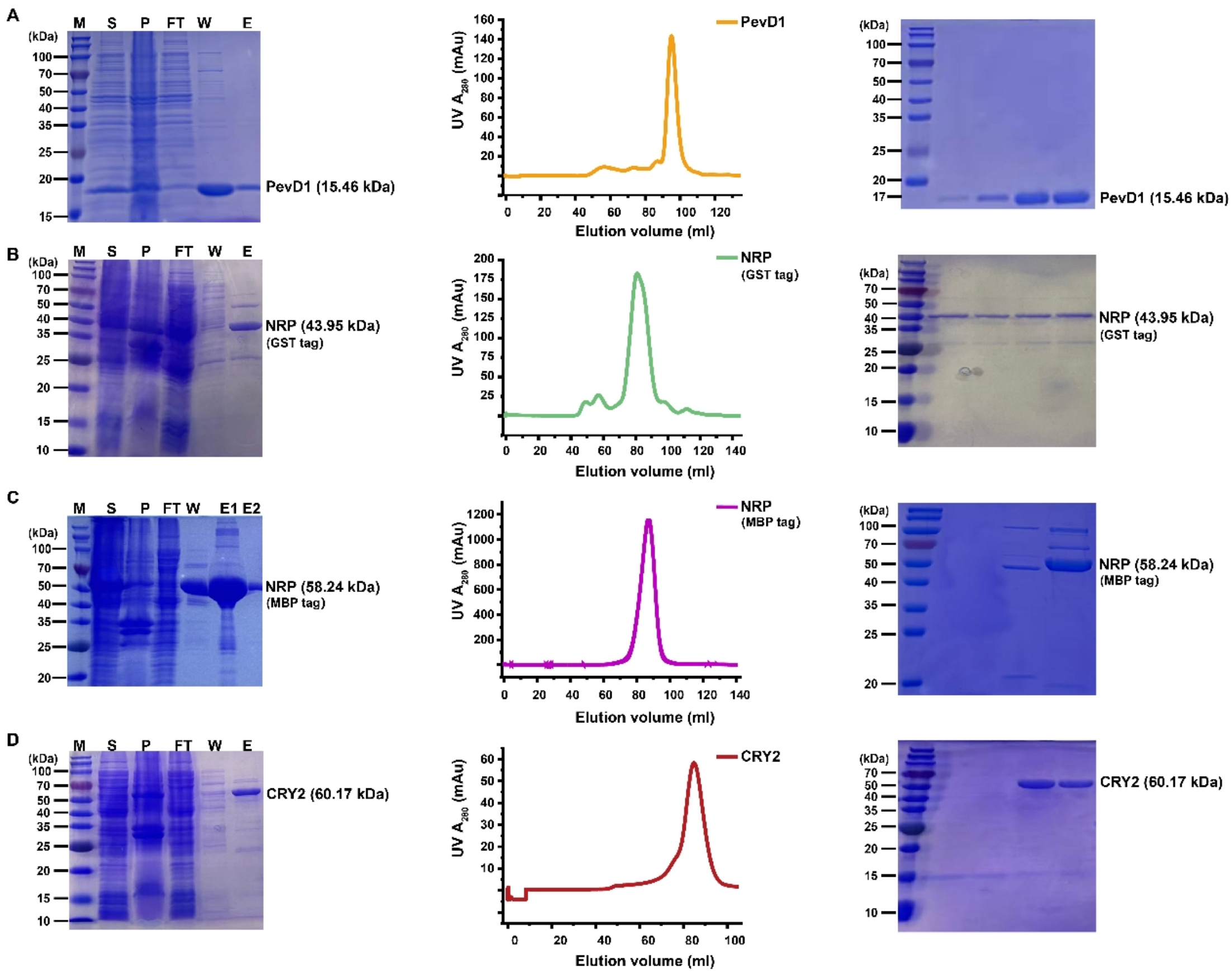

Figure S4. Expression and purification of PevD1, GST-NRP, MBP-NRP, and CRY2 proteins. The SDS–PAGE analysis and size-exclusion chromatography profiles of PevD1 (A), GST-NRP (B), MBP-NRP (C), and CRY2 (D). The left panels show SDS-PAGE analysis of samples collected during the purification process, including the supernatant (S), pellet (P), flow-through fraction (FT), wash fraction (W), and elution fractions (E1-E2). The right panels present the corresponding size-exclusion chromatography profiles. SDS-PAGE analysis of the major peak fractions from the size-exclusion chromatography revealed a single predominant band at the expected molecular weight, indicating high purity of the purified proteins.

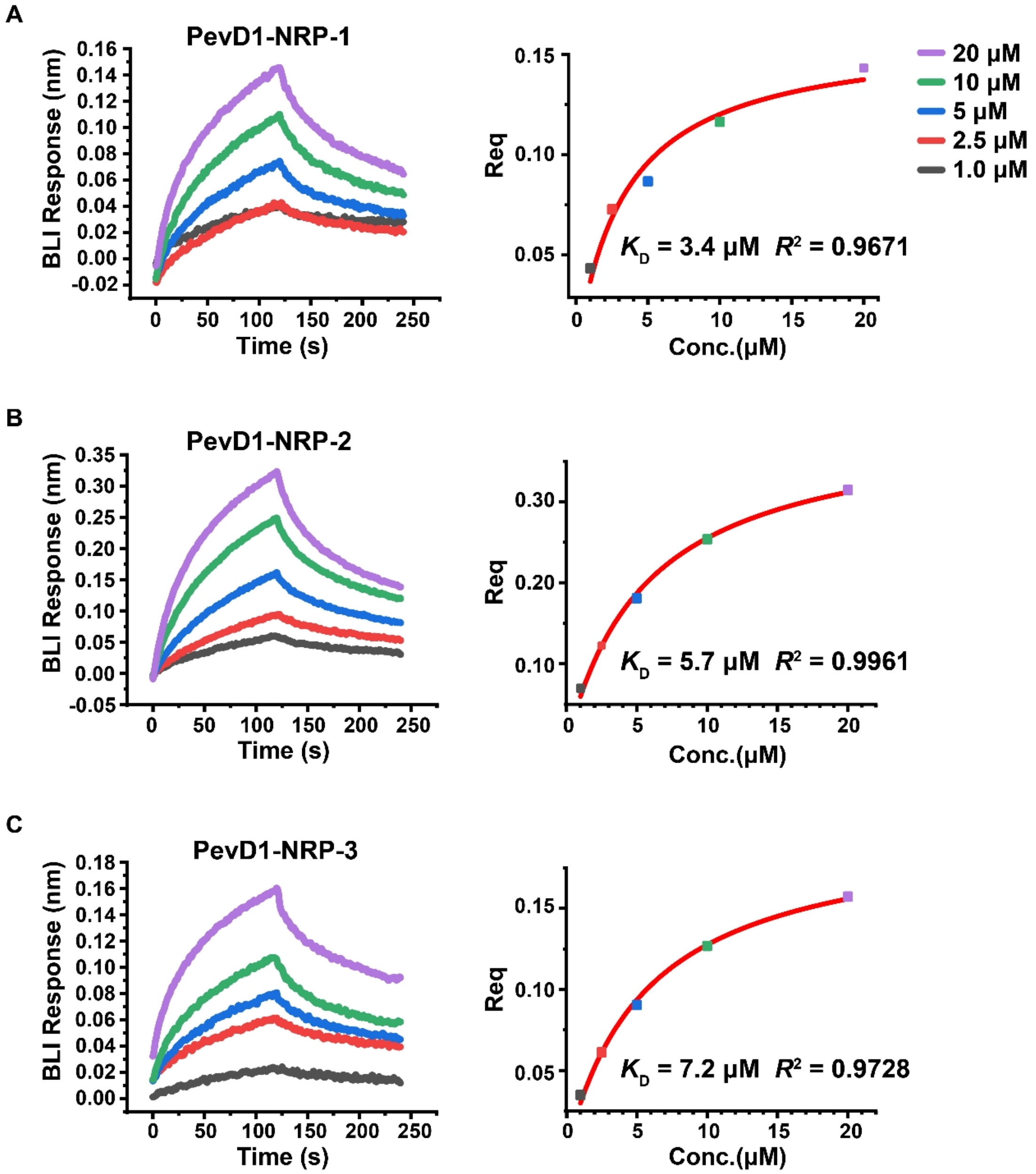

Figure S5. Quantitative analysis of the molecular binding characteristics between PevD1 and NRP using Bio-Layer Interferometry. (A-C) The binding kinetics of the two proteins were assessed at different gradient concentrations (20 µM, 10 µM, 5 µM, 2.5 µM, 1 µM). The K_D values ranged from 3.4 µM to 7.2 µM across three independent experiments.

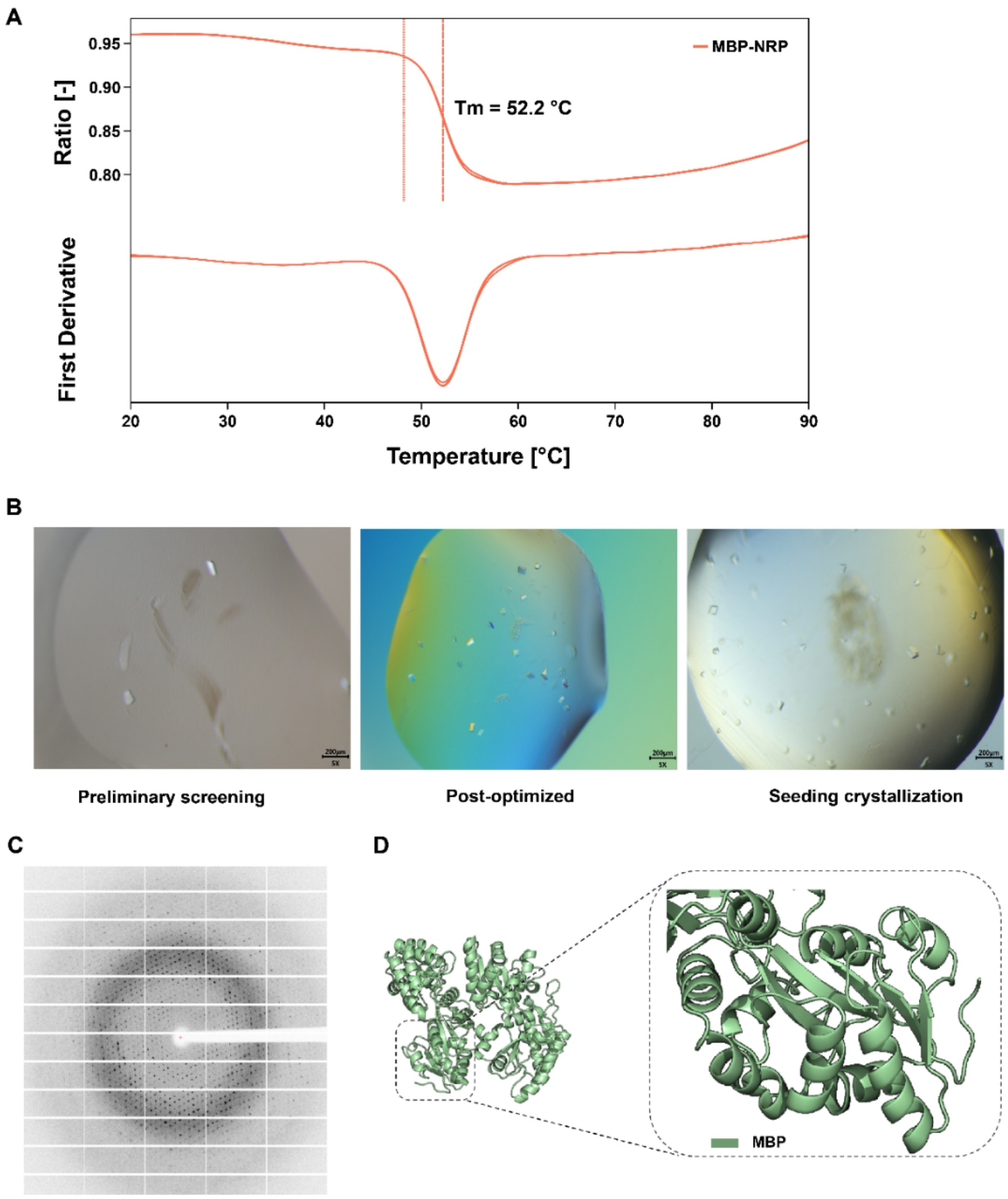

Figure S6. Crystallographic study of the NRP protein. (A) The melting temperature of MBP tagged NRP protein tested by DSF. **(B)** Crystal images of MBP tagged NRP protein obtained in preliminary screening, post-optimized and seeding crystallization conditions. **(C)** The diffraction image of MBP tagged NRP protein with highest resolution at 2.0 Å. **(D)** The predicted MBP tagged NRP protein structure using AlphaFold3, with an enlarged view showing the structure of MBP.

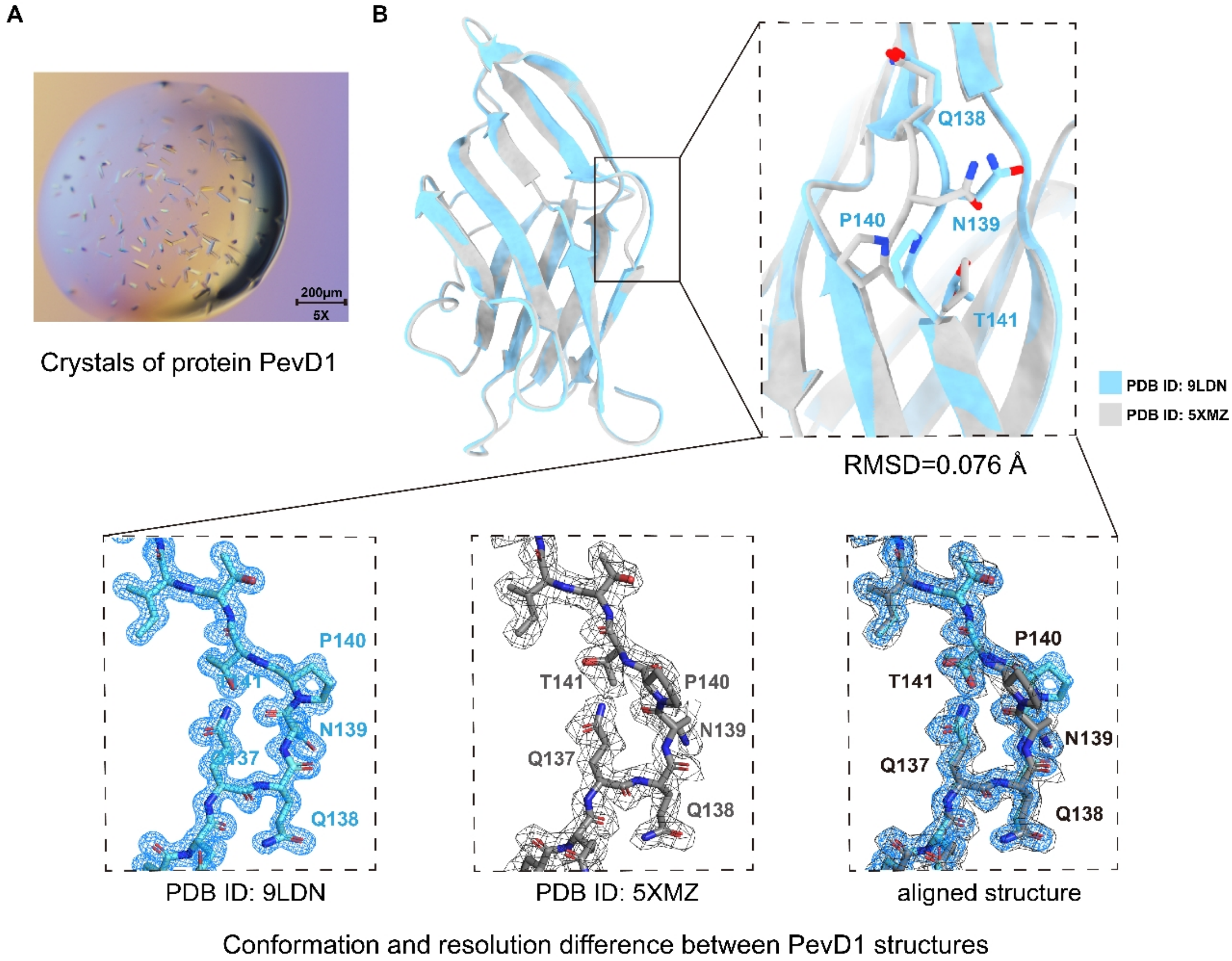

Figure S7. Crystal structure determination of PevD1. (A) Representative crystals of the *apo* PevD1. **(B)** Structural superposition of PevD1 (light sky blue) with the previously reported structure (PDB ID: 5XMZ, gray) reveals conformational changes at residues Q138, N139, and P140. Electron density maps and structural comparison of PevD1 are shown below.

Table S1. Primer sequences used for site-directed mutagenesis of PevD1.

Index	Primer Name	Primer Sequence
F1	D31_Fw	GCGCCTCCGGCGCCGAATATGTATGA
R1	D31_Rv	CGCCGGAGGCGCACCG
F2	E108_Fw	GCGTTGGGACTGGCTGTCGG
R2	E108_Rv	CAGTCCCAACGCGTGATACAGGCTC
F3	L111_Fw	GGCGCTGTCGGTTTCTACGGC
R3	L111_Rv	ACCGACAGCGCCTCCCAACTCGTGAT
F4	D150_Fw	GCGGCACCAGCGGAGGC
R4	D150_Rv	CGCTGGTGCCGCGGGCAGAGAGTCGATCACG
F5	R90_Fw	AAATACGCATTTGCGTTGTCTTCCGGTAAGCAATACG
R5	R90_Rv	AACGCAAATGCGTATTTGCTCTCACCGCACGTGATAACG
F6	Y106_Fw	GCGCACGCGTTGGGACTGGC
R6	Y106_Rv	CAACGCGTGCGCCAGGCTCAGTGCAAATTCGTATTGC

Table S2. All replicate SPR datasets for the PevD1–NRP, NRP–CRY2 and A6–PevD1 interaction analyses.

K_D values determined by SPR assay			
	Test 1	Test 2	Test 3
PevD1-NRP	0.19 μM	0.50 μM	0.56 μM
NRP-CRY2	4.34 μM	5.59 μM	-
A6-PevD1	35.30 μM	32.60 μM	44.10 μM

Table S3. Crystallography data collection and refinement statistics.

	**PevD1**
**PDB ID**	9LDN
Space Group	*C 2 2 2_1*
Cell Dimension: a (Å)	58.41
b (Å)	79.59
c (Å)	55.28
Wavelength (Å)	0.9875
Reflections (unique)	28653 (2815)
Resolution Range (Å)	35.85- 1.35
Highest-Resolution Shell (Å)	1.398 - 1.350
Redundancy	12.52
I/σ (I)	4.70
Highest-Resolution Shell $CC_{1/2}$	0.9039
Completeness (%)	100.00
Rwork/Rfree	0.220/0.239
**RMS Values**	
Bond length (Å)	0.006
Bond angle (°)	0.95
**Numbers of Non-hydrogen Atoms**	
Protein	993
Inhibitor	
Water Oxygen	61
Clashscore	2.21
MolProbity Score	1.00
**Ramachandran plot**	
Favored (%)	100.00
Allowed (%)	0.00
Outliers (%)	0.00

Table S4. **Candidate compounds information discovered by virtual screening.**

Compound	Structure	Docking Score (kcal/mol)	SPECS No.
A6	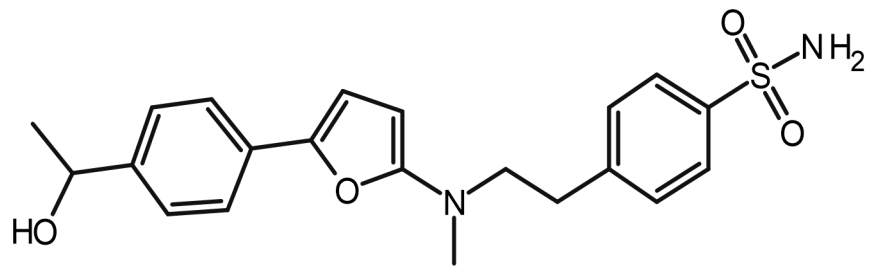	-5.379	AN-465/43411507
A29	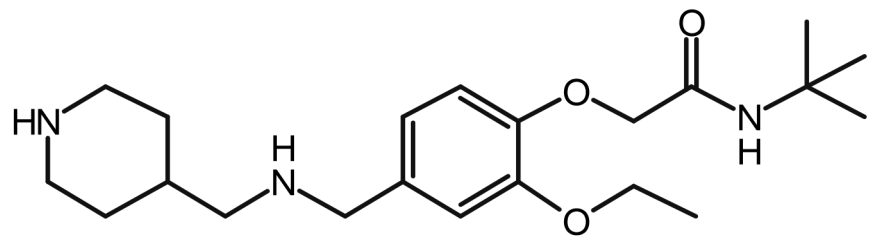	-5.254	AN-465/43384171
A34	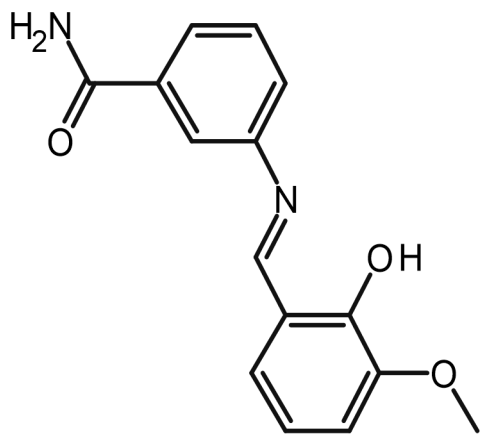	-5.168	AN-329/11481427
A11	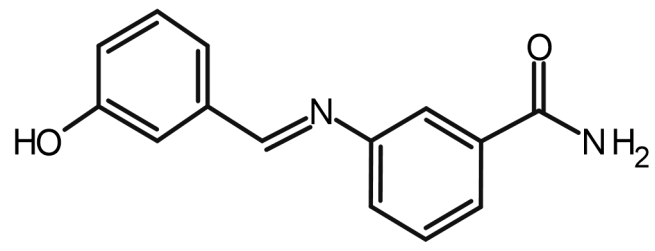	-5.154	AN-329/10737012
A13	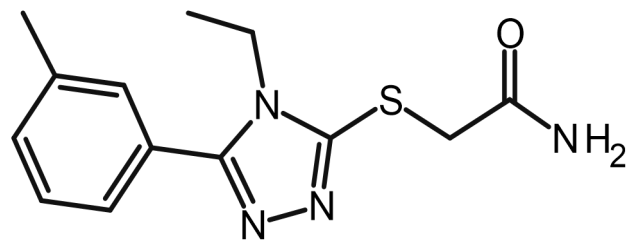	-5.150	AG-690/40753252
A8	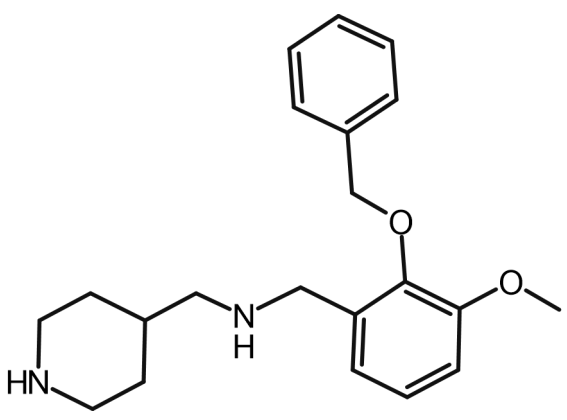	-5.032	AN-465/43384144
A49	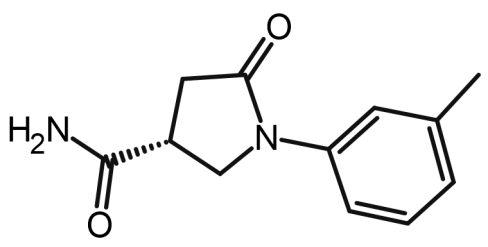	-5.026	AN-329/11481422
A12	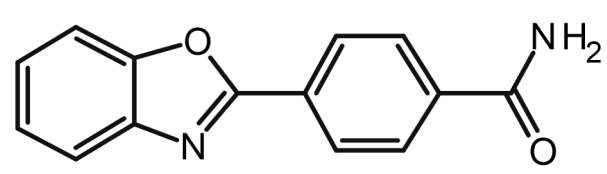	-4.928	AP-970/43482576

Compound	Structure	Docking Score (kcal/mol)	SPECS No.
A22	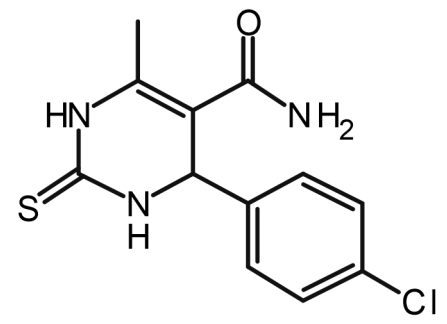	-4.910	AN-023/13711036
A37	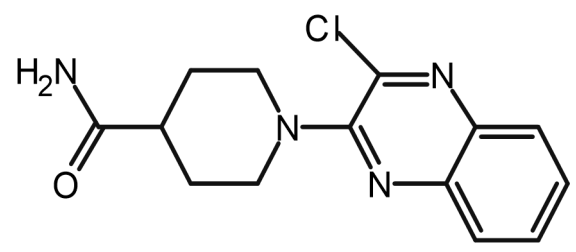	-4.827	AP-970/43482408
A43	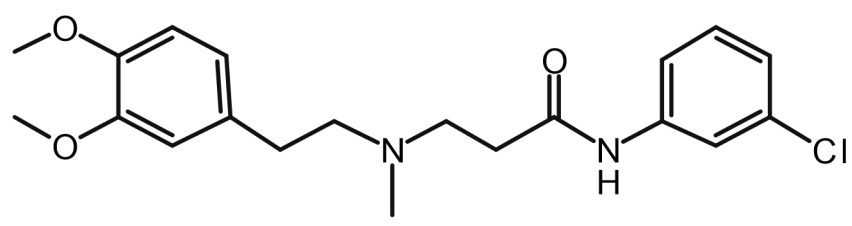	-4.735	AT-057/43468920
A33	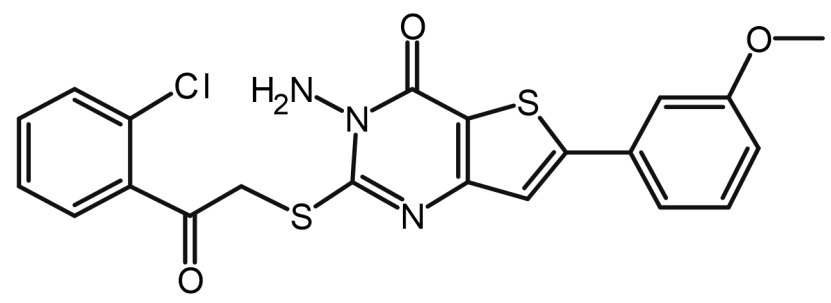	-4.700	AO-476/43415661
A2	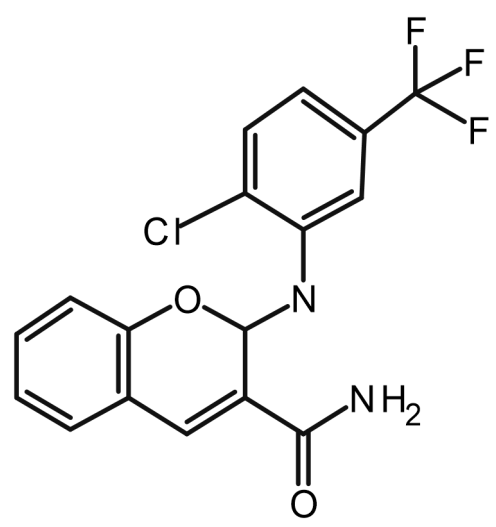	-4.653	AK-968/15361666
A14	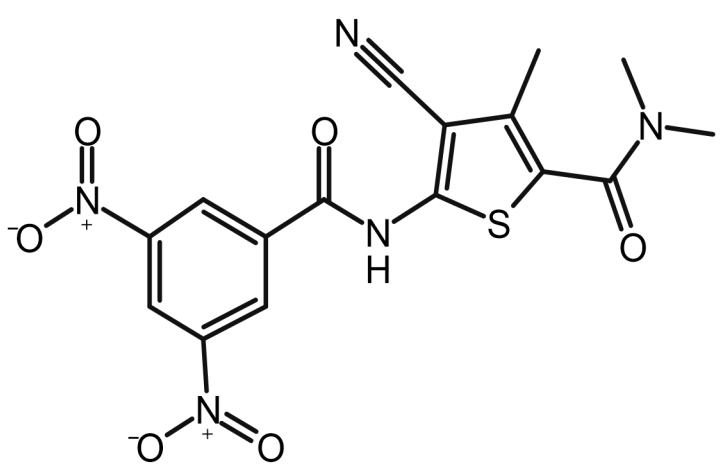	-4.514	AK-968/40709524
A38	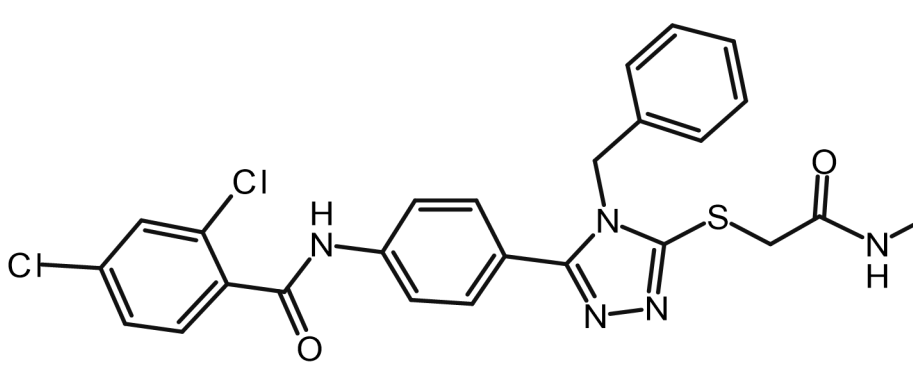	-4.465	AQ-088/41468747
A30	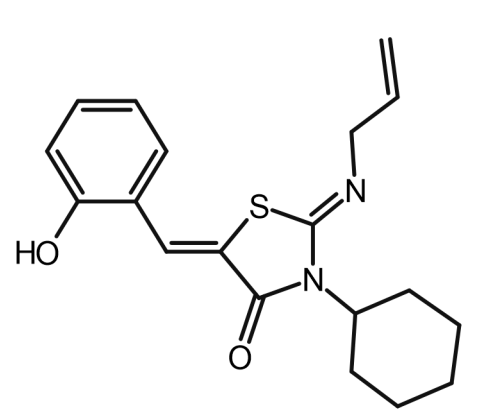	-4.429	AG-670/41998516

Compound	Structure	Docking Score (kcal/mol)	SPECS No.
A48	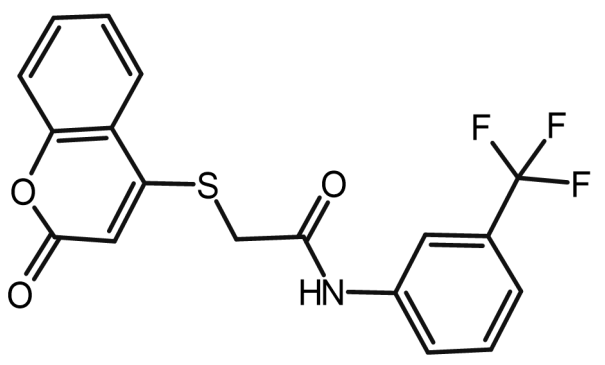	-4.392	AI-204/31717060
A42	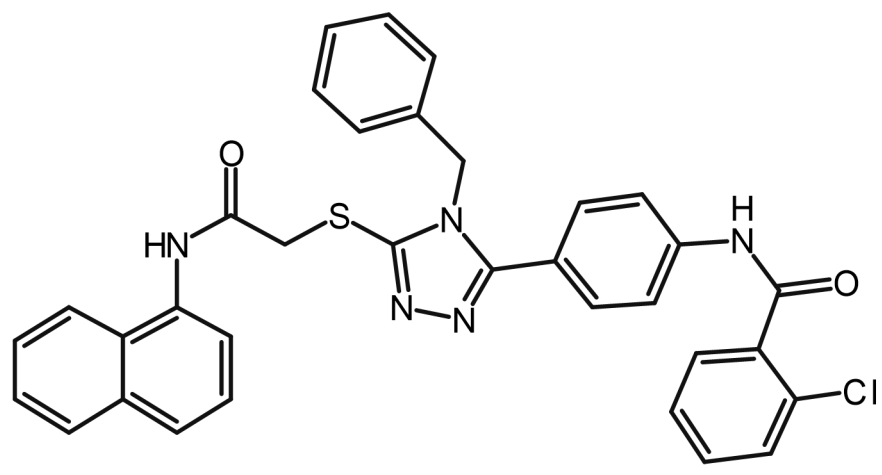	-4.353	AQ-088/42013797
A45	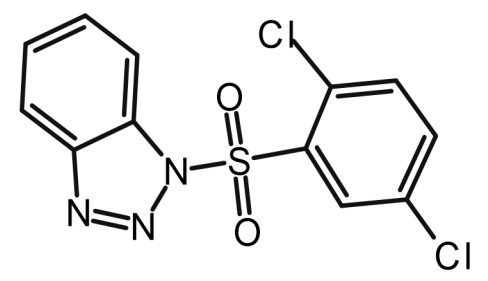	-4.254	AG-205/36915223
A19	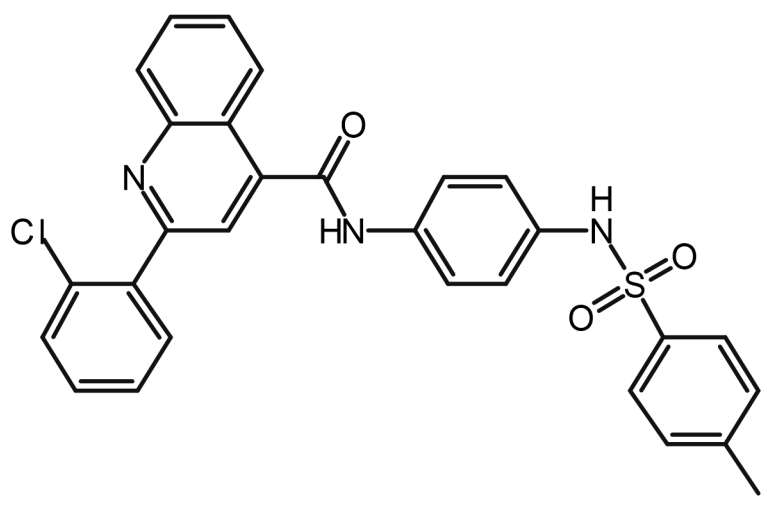	-4.249	AK-968/11992808
A27	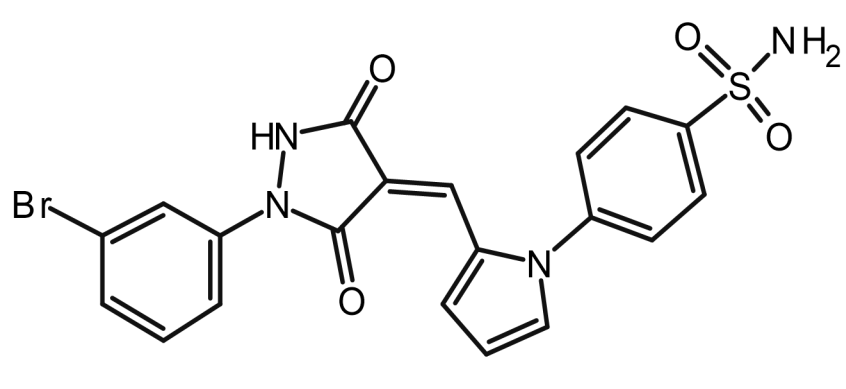	-4.211	AN-465/15152242
A36	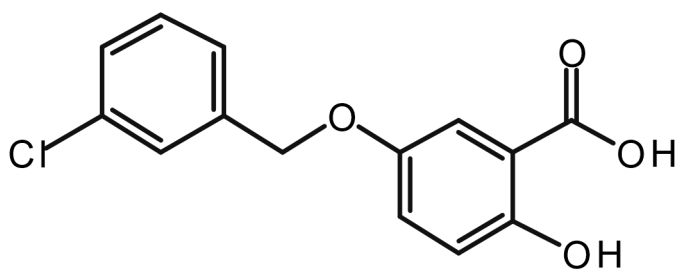	-4.141	AP-970/43507355
A17	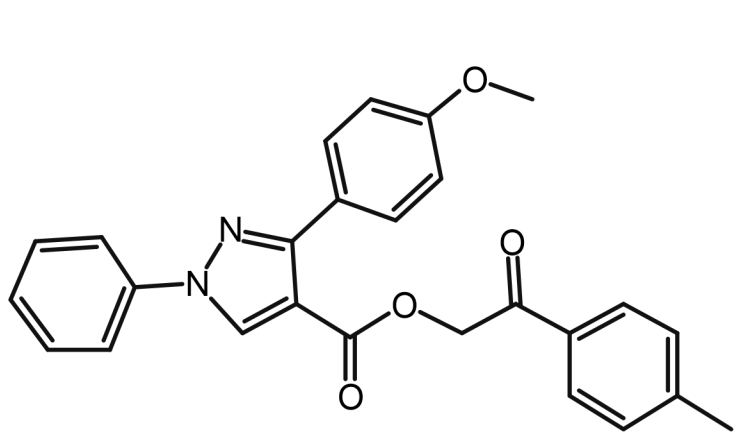	-4.121	AK-918/40706123
A32	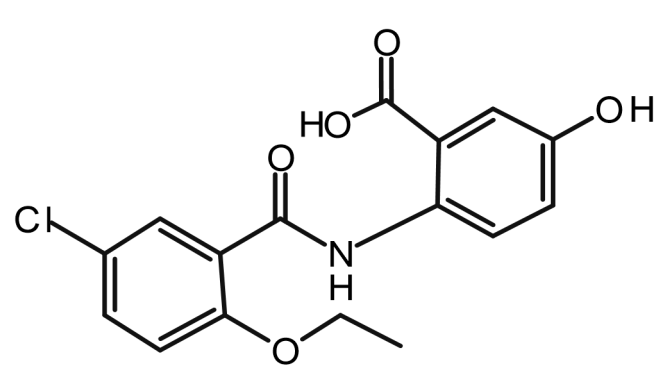	-4.092	AN-979/42723138

Compound	Structure	Docking Score (kcal/mol)	SPECS No.
A40	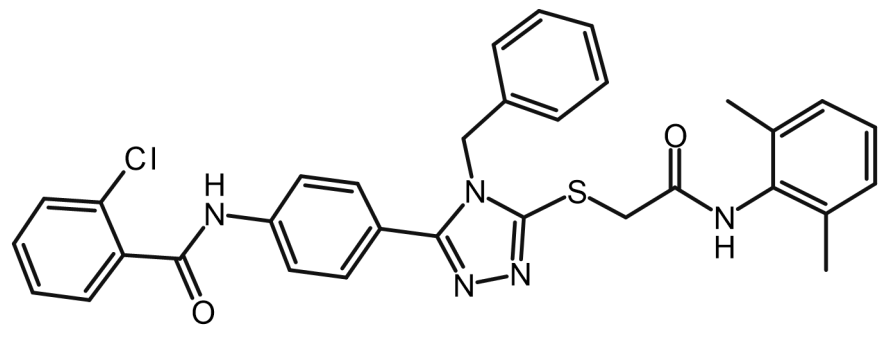	-4.092	AQ-088/41468806
A46	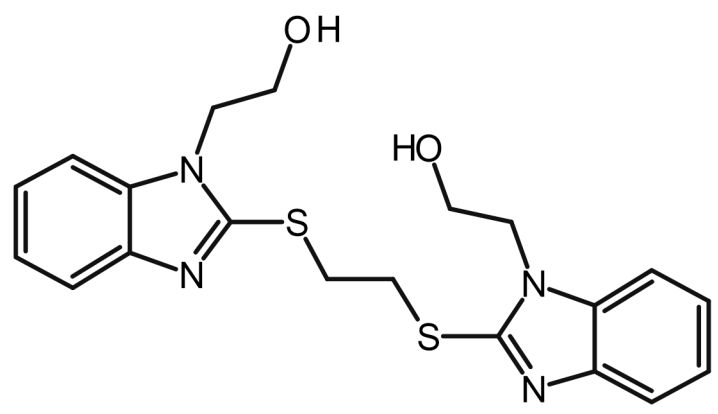	-4.067	AG-670/11899358
A21	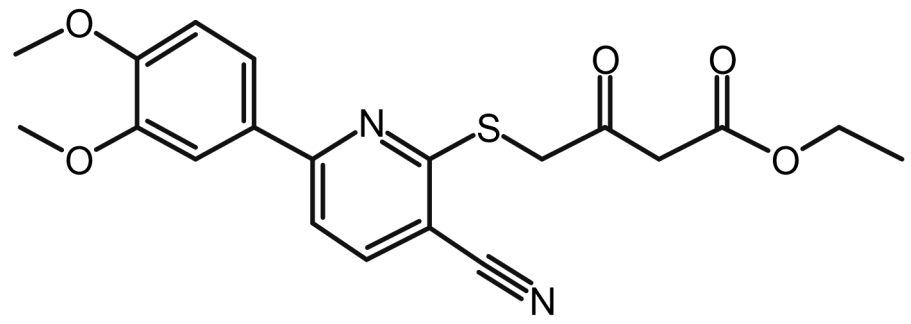	-4.055	AM-807/12740175
A10	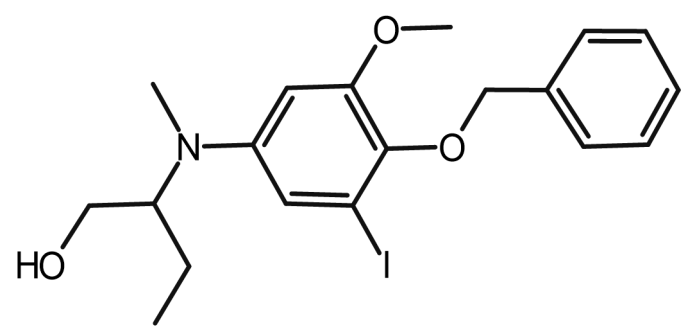	-4.052	AP-970/42897940
A35	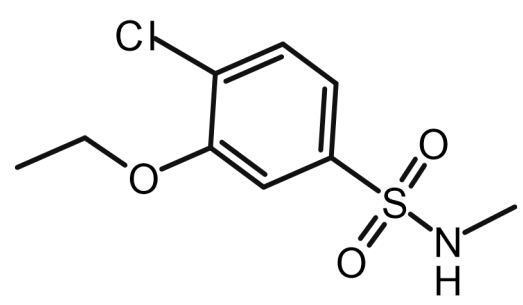	-4.016	AP-263/43502651
A20	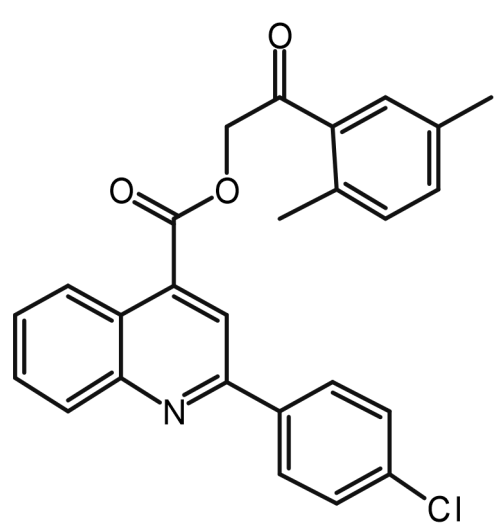	-4.008	AK-968/13072047
A26	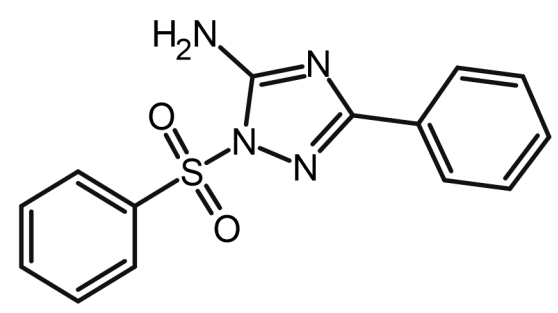	-3.911	AE-848/42278410
A18	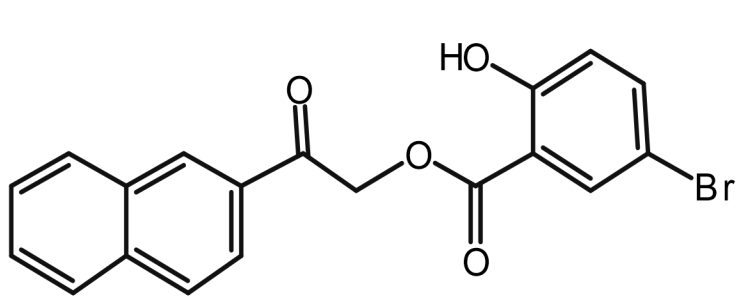	-3.909	AK-918/41944947

Compound	Structure	Docking Score (kcal/mol)	SPECS No.
A15	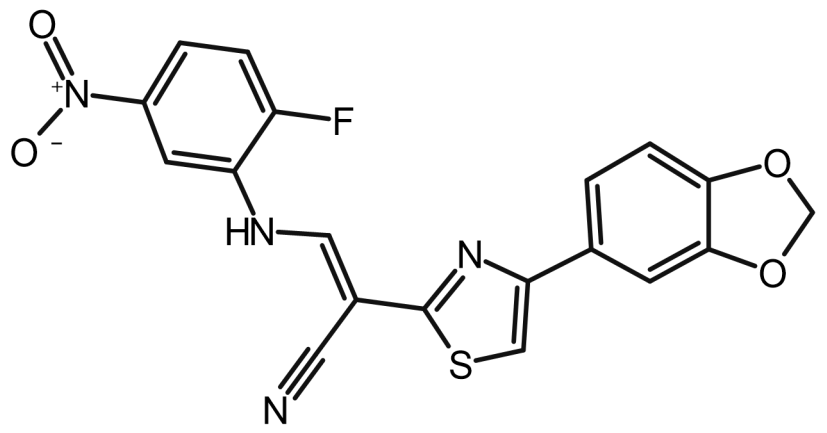	-3.684	AE-848/15341257
A16	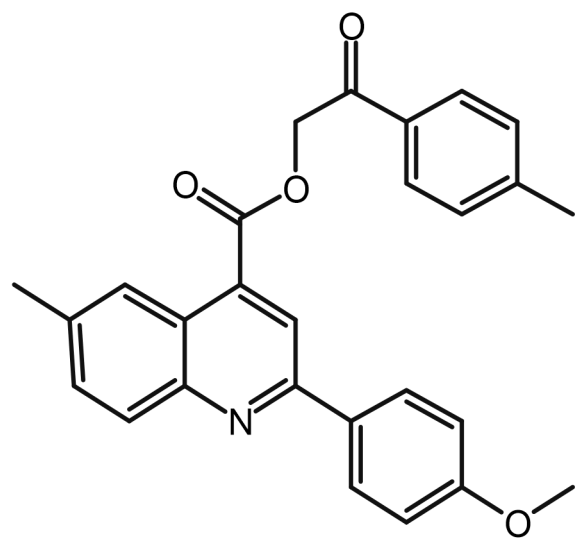	-3.682	AK-918/13281503
A47	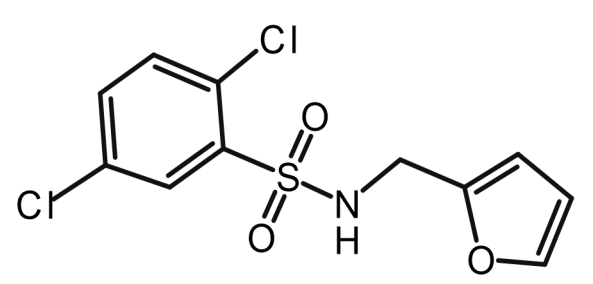	-3.627	AG-690/11633971
A3	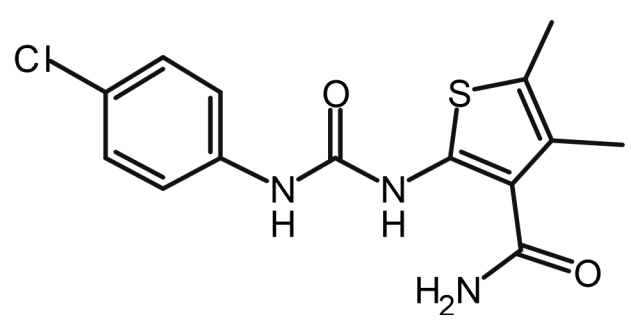	-3.619	AN-329/40366623
A50	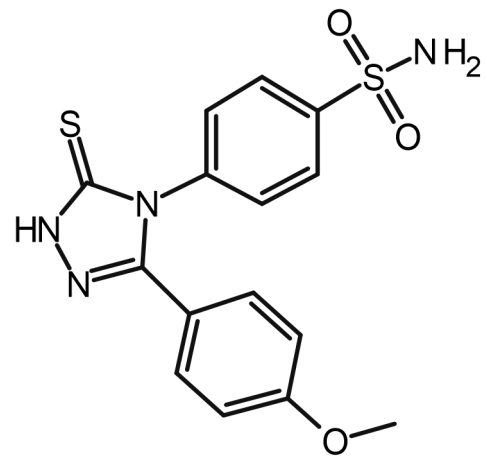	-3.555	AJ-292/41695020
A31	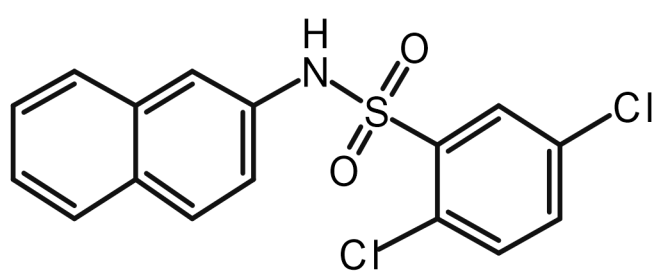	-3.495	AN-652/13035575
A23	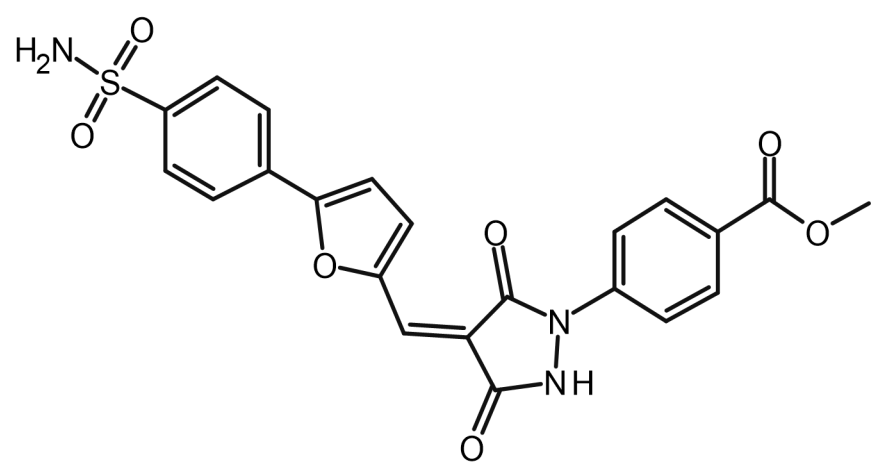	-3.343	AN-023/15593034

Compound	Structure	Docking Score (kcal/mol)	SPECS No.
A24	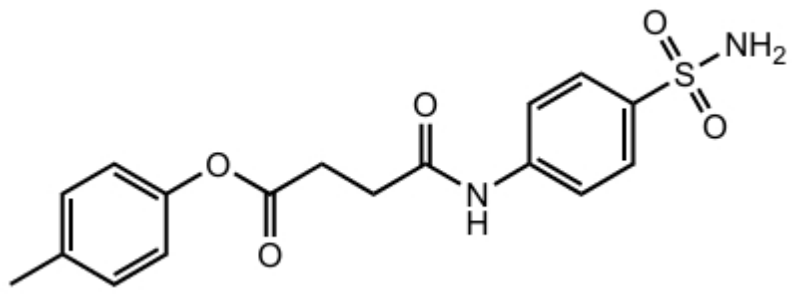	-3.274	AN-329/40805572
A28	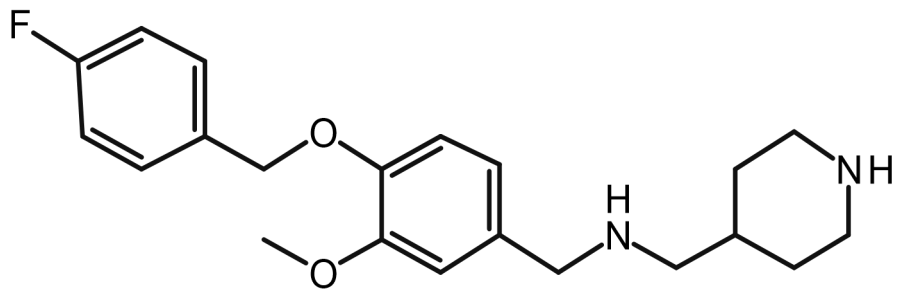	-3.236	AN-465/43384075
A4	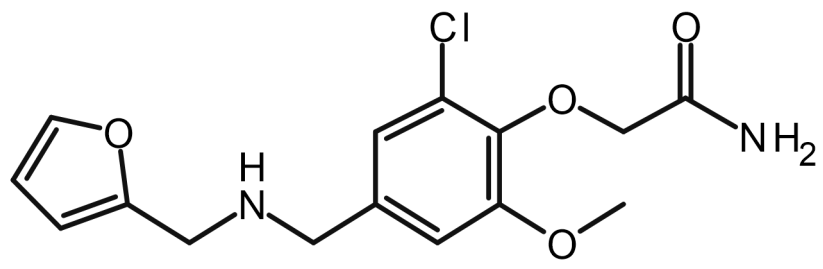	-3.229	AN-465/43013022
A7	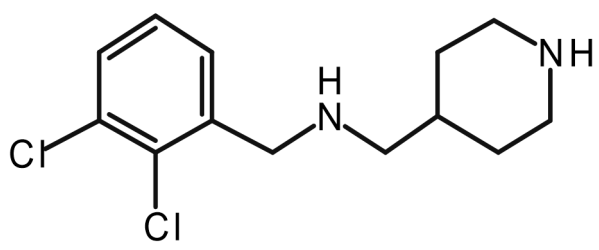	-2.996	AN-465/43384057
A44	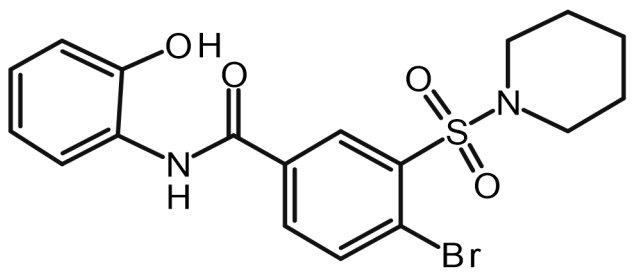	-2.775	AK-918/11144033
A25	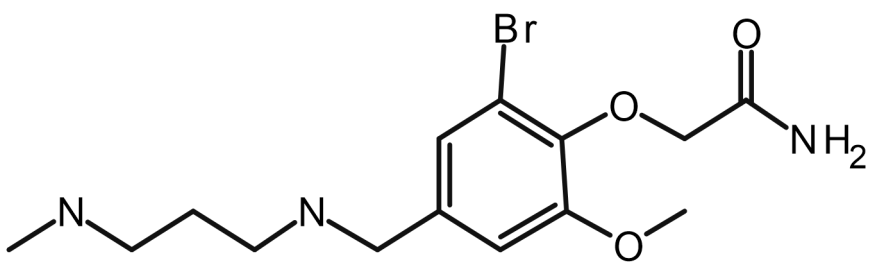	-2.763	AN-465/42887164
A9	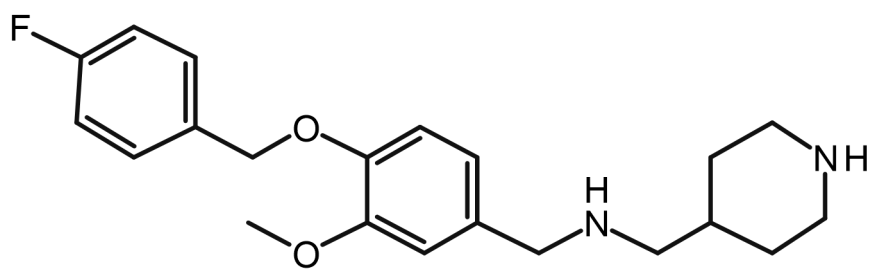	-2.711	AN-465/43384150
A5	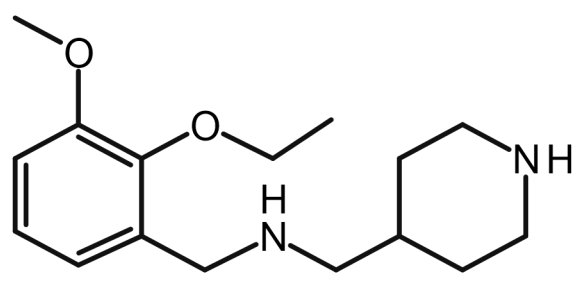	-2.652	AN-465/43384034
A1	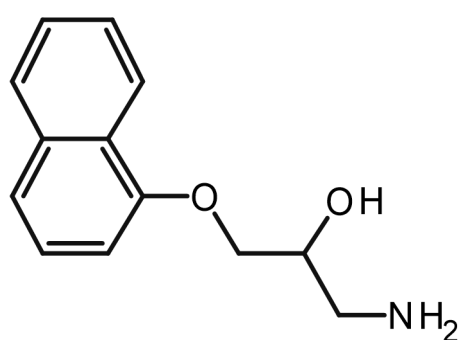	-2.399	AG-777/36176008

Compound	Structure	Docking Score (kcal/mol)	SPECS No.
A39	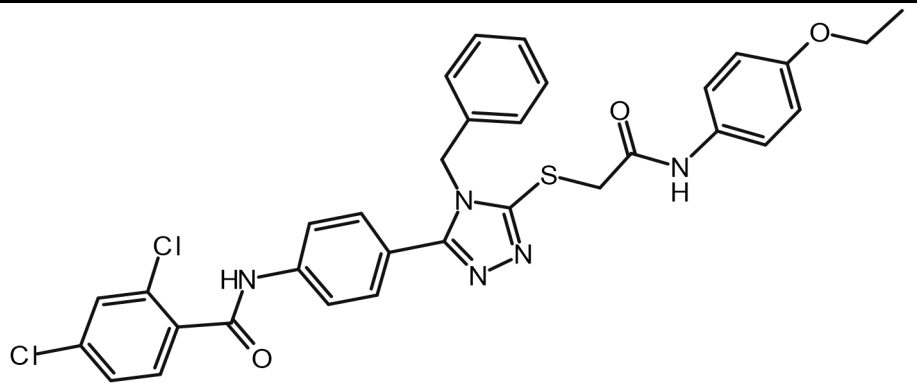	-1.703	AQ-088/41468756
A41	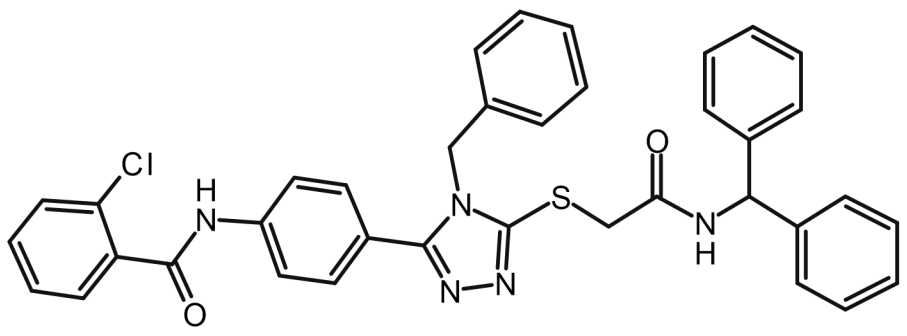	-1.358	AQ-088/41468813
